# Nerve Fiber Flux Analysis Using Wide-Field Swept-Source Optical Coherence Tomography

**DOI:** 10.1167/tvst.7.1.16

**Published:** 2018-02-07

**Authors:** Ou Tan, Liang Liu, Li Liu, David Huang

**Affiliations:** 1 Casey Eye Institute, Oregon Health and Science University, Portland, OR, USA

**Keywords:** nerve fiber trajectory, optical coherence tomography, nerve fiber layer thickness map, nerve fiber flux

## Abstract

**Purpose:**

To devise a method to quantify nerve fibers over their arcuate courses over an extended peripapillary area using optical coherence tomography (OCT).

**Methods:**

Participants were imaged with 8 × 8-mm volumetric OCT scans centered at the optic disc. A new quantity, nerve fiber flux (NFF), represents the cross-sectional area transected perpendicular to the nerve fibers. The peripapillary area was divided into 64 tracks with equal flux. An iterative algorithm traced the trajectory of the tracks assuming that the relative distribution of the NFF was conserved with compensation for fiber connections to ganglion cells on the macular side. Average trajectory was averaged from normal eyes and use to calculate the NFF maps for glaucomatous eyes. The NFF maps were divided into eight sectors that correspond to visual field regions.

**Results:**

There were 24 healthy and 10 glaucomatous eyes enrolled. The algorithm converged on similar patterns of NFL tracks for all healthy eyes. In glaucomatous eyes, NFF correlated with visual field sensitivity in the arcuate sectors (Spearman ρ = 0.53–0.62). Focal nerve fiber loss in glaucomatous eyes appeared as uniform tracks of NFF defects that followed the expected arcuate fiber trajectory.

**Conclusions:**

Using an algorithm based on the conservation of flux, we derived nerve fiber trajectories in the peripapillary area. The NFF map is useful for the visualization of focal defects and quantification of sector nerve fiber loss from wide-area volumetric OCT scans.

**Translational Relevance:**

NFF provides a cumulative measure of volumetric loss along nerve fiber tracks and could improve the detection of focal glaucoma damage.

## Introduction

Retinal nerve fiber layer (NFL) thinning is widely recognized as a sign of glaucoma and other optic neuropathies. In standard optical coherence tomography (OCT) evaluations of glaucoma, the NFL thickness profile is typically measured using a cylindric cross-sectional scan of 3.4-mm diameter around the optic disc.^1,2^ In clinical diagnosis, the NFL thickness profile at different angular positions or sectors is compared with an age-matched healthy reference. Although this approach has good diagnostic accuracy,^3–11^ it relies on a single cross-sectional scan, and the results could be significantly affected by even a small segmentation error or anatomic anomaly (e.g., vessel crossing, epiretinal membrane).

With the improvement of OCT scan speed, volumetric scans over wider areas can be performed in short times, and eye motion error can be corrected using real-time eye tracking or registration during image post processing. Reliable NFL mapping over a 5- to 6-mm diameter around the optic disc is already provided in most commercial OCT instruments. Volumetric analysis of the NFL thickness in pie-shaped sectors is more repeatable than in the classic 3.4-mm diameter profile.^12–15^ Volumetric analyses usually compare values at the same location of the two-dimensional thickness or focal loss map, or by using super pixel analysis^16–18^; however, the information cannot be combined along the nerve fiber bundle.

**Figure 1:**
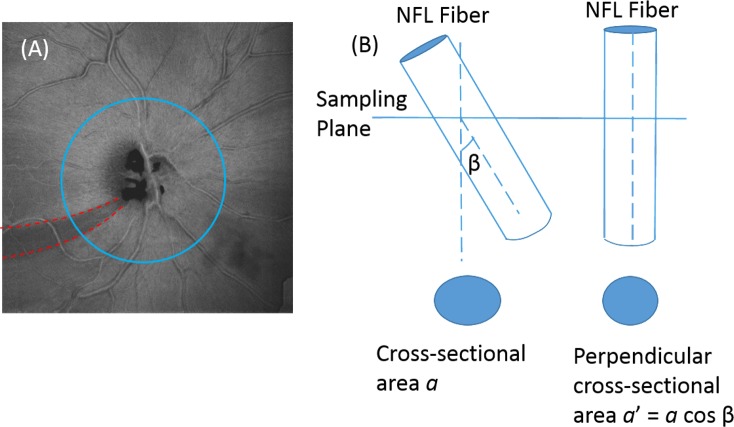
Flux analysis conserves the skew-corrected NFL cross-sectional area integrated along a circular profile. (A) Average reflectance in peripapillary NFL of a glaucomatous eye showing the arcuate course of a bundle defect (red dashed lines). Sampling circles (i) cut across nerve fibers at a skewed (nonperpendicular) angle β. (B) An oblique cut across a nerve fiber increases the cross-sectional area. Flux analysis multiplies the apparent cross-sectional area by the cosine of angle β, recovering the perpendicular cross-sectional area.

One complication in NFL map analysis is that nerve fiber bundles do not exactly follow radial lines from the optic disc, but rather arcs toward and around the macula. The superior and inferior arcuate bundles are significantly off radial in direction, and cylindrical profile measurements could be expected to vary greatly with the distance from the optic disc. One source of variation is that the same angular position samples different fibers at different sampling circle diameters, and the same nerve fiber bundle can traverse through more than one pie-shaped sector. Another problem is that a cylindric section cuts through arcuate bundles at nonperpendicular angles, and the resulting NFL cross-sectional area becomes larger due to the nonperpendicularity (Fig. 1). These two problems make it more difficult to visualize subtle nerve fiber bundle defects and quantify volumetric loss of nerve fiber tissue in sectors that correlate with the visual field (VF).

In this paper, we develop a new method to address these two problems using the mathematics of flux analysis, which is well developed in field theory. In Gauss's law for electric field, he formulated the flux theorem to relate electric charges to flux (electric field lines). The field lines connect positive and negative charges, and the conservation of charge could be shown to be equivalent to the conservation of flux. In our analysis, retinal ganglion cells (RGC) could be thought of as positive charges, nerve fibers as flux lines, and the optic nerve head (ONH) as a collective sink of negative charges. The number of ganglion cells and nerve fibers are equal and constant (conserved) in each eye. Using the assumption that the cross-sectional area of nerve fibers, when cut at a perpendicular angle, are approximately conserved in the area close to the optic disc, the flux analysis can trace the trajectory of nerve fiber bundles and measure them over a wide-area map. We demonstrate the feasibility of this approach using wide-field volumetric scans of the NFL with a swept-source OCT system. The nerve fiber flux (NFF), which is defined as the perpendicular cross-sectional area of the nerve fibers, is not affected by variation in the crossing angle between the sampling circle and the nerve fibers. Therefore, the NFF should be better conserved over a wide range of sampling circle diameters. Using the principle of conservation of flux, we derived nerve fiber track trajectories in a group of healthy reference human subjects. The NFF map was then divided into sectors that correspond to VF regions for correlation analysis in glaucoma patients.

## Methods

### Participants

The research protocol was approved by the institutional review boards at Oregon Health and Science University (OHSU) and carried out in accordance with the tenets of the Declaration of Helsinki. Written informed consent was obtained from each participant after explanation of the nature of the study. Participants were recruited at the Casey Eye Institute/OHSU according to the “Functional and Structural OCT in Glaucoma” study protocol (https://clinicaltrials.gov/ct2/show/NCT01957267).

Briefly, healthy control participants met the following criteria in both eyes: intraocular pressures less than 21 mm Hg and a normal Humphrey visual field (HVF) on standard achromatic perimetry by the Swedish Interactive Threshold Algorithm 24-2 testing program (HFA II; Carl Zeiss Meditec, Inc., Dublin, CA) with mean deviation (MD), glaucoma hemifield test (GHT), and pattern standard deviation (PSD) within normal limits. In addition, healthy subjects had a normal appearing ONH and NFL on ophthalmoscopic examination and an open angle determined by gonioscopy.

The criteria for inclusion of glaucoma participants were a glaucomatous ONH appearance on examination and NFL defects with HVF defects with PSD or GHT outside normal limits (*P* < 0.05 and *P* < 1%, respectively) in a consistent pattern on two qualifying HVF exams. Exclusion criteria for both groups included vision less than 20/40, age between 40 and 79 years at enrollment, any ocular surgery other than cataract extraction, other diseases that might cause HVF or ONH abnormality, and factors that might preclude the participant from performing the study procedures or completing the study.

### The OCT System and Scan

A 100-kHz swept-source OCT prototype was used to collect the OCT scans.^19^ The device used a short-cavity tunable laser (Axun, Inc., Billerica, MA) operating at a central wavelength of 1050 nm. The resolution was 5.3-μm axially and 18-μm laterally at an imaging depth of 2.9 mm in tissue. The ocular light power exposure was 1.9 mW, which was within the American National Standards Institute safety limit.

For each participant, only one eye was scanned, obtaining both disc and macular scans. The eye was scanned using an 8 × 8-mm high-density raster scan pattern, taking approximately 4.3 seconds. Each volume contained 640 cross-section images, or B-frames. Each B-frame contained 640 axial lines. The scan protocol included four scans consisting of two horizontal and two vertical scans. To remove eye motion artifacts, an orthogonal registration algorithm was applied to register and merge all four scans.^20^

### Nerve Fiber Layer Thickness

**Figure 2:**
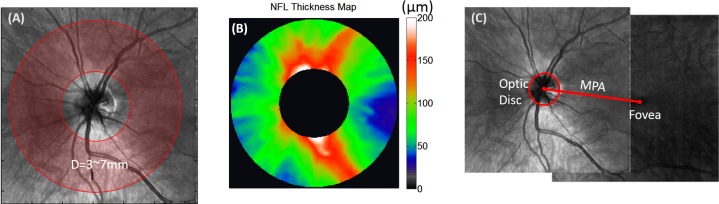
NFL thickness map and MPA detection. (A) Resampling of 21 concentric circles with diameters of 3.0 to 7.0 mm from an 8 × 8-mm OCT scan. (B) NFL thickness map reconstructed from the 21 circles. (C) The angle of the MPA (red line) was measured on the registered en face OCT image of the macular and disc regions.

The disc volume scan was processed to obtain an en face OCT image (full depth) and a NFL thickness map. On the en face OCT image, the disc boundary was marked by coauthor LL with an ellipse (Fig. 2). Concentric cylindrical cross sections of varying radii were resampled from the volume scan (Fig. 2) and upsampled to 1024 transverse points by interpolation. We selected 21 rings with radii from 1.5 to 3.5 mm for analysis. The NFL thickness was measured between the inner limiting membrane (ILM) and the outer NFL boundary, which were segmented using a modified version of our previously described computer algorithm.^21^ Coauthor LL validated the segmentation results on the OCT B-frame of each ring and corrected the boundary if needed.

To obtain “true” NFL thickness perpendicular to inner retinal surface, the NFL thickness was corrected by the cosine of the angle between the normal of the ILM elevation map and the OCT axial line.

The axial eye length was used to correct optical magnification variation by adjusting the transverse scan dimensions of the OCT images with a previously described method.^22,23^ The axial length was measured using an IOLMaster 500 (Zeiss Meditec, Inc., Dublin, CA). The resulting NFL map was then used for flux analysis.

### Maculopapillary Axis

The disc and macular scans are registered and montaged to measure the orientation of the maculopapillary axis (MPA, Fig. 2). The computer calculated the center of the disc as the geometric center of the manually marked disc boundary, and the foveal center was calculated as the center of the foveal thin spot on the retinal thickness map. The MPA was drawn between the fovea and disc centers. The MPA angle was defined as the angle between the MPA and the horizontal line.

### Calculation of the NFL Trajectory Map Using Flux Analysis

Nerve fibers can be tracked along cylindric profiles of increasing radii assuming that the perpendicular cross-sectional area (including associated glial tissue) is approximately conserved, and that relatively few fibers terminate in RGC in the peripapillary area. The peripapillary cylindrical OCT cross-sections transect all nerve fibers converging on the disc, but generally at a skewed angle *β* that deviates from the perpendicular. To recover the perpendicular cross-section of the entire NFL, we performed a circle line integral of the NFL thickness *T* after correcting for cosine:

\newcommand{\bialpha}{\boldsymbol{\alpha}}\newcommand{\bibeta}{\boldsymbol{\beta}}\newcommand{\bigamma}{\boldsymbol{\gamma}}\newcommand{\bidelta}{\boldsymbol{\delta}}\newcommand{\bivarepsilon}{\boldsymbol{\varepsilon}}\newcommand{\bizeta}{\boldsymbol{\zeta}}\newcommand{\bieta}{\boldsymbol{\eta}}\newcommand{\bitheta}{\boldsymbol{\theta}}\newcommand{\biiota}{\boldsymbol{\iota}}\newcommand{\bikappa}{\boldsymbol{\kappa}}\newcommand{\bilambda}{\boldsymbol{\lambda}}\newcommand{\bimu}{\boldsymbol{\mu}}\newcommand{\binu}{\boldsymbol{\nu}}\newcommand{\bixi}{\boldsymbol{\xi}}\newcommand{\biomicron}{\boldsymbol{\micron}}\newcommand{\bipi}{\boldsymbol{\pi}}\newcommand{\birho}{\boldsymbol{\rho}}\newcommand{\bisigma}{\boldsymbol{\sigma}}\newcommand{\bitau}{\boldsymbol{\tau}}\newcommand{\biupsilon}{\boldsymbol{\upsilon}}\newcommand{\biphi}{\boldsymbol{\phi}}\newcommand{\bichi}{\boldsymbol{\chi}}\newcommand{\bipsi}{\boldsymbol{\psi}}\newcommand{\biomega}{\boldsymbol{\omega}}\begin{equation}\tag{1}\mathop \oint \nolimits T\cos \beta \,dl{\rm }\end{equation}

where *dl* is the differential length element along the circle.

In this study, because the sampling circles were concentric rings around the disc center, we used polar coordinates that enabled Equation 1 to be rewritten as

\newcommand{\bialpha}{\boldsymbol{\alpha}}\newcommand{\bibeta}{\boldsymbol{\beta}}\newcommand{\bigamma}{\boldsymbol{\gamma}}\newcommand{\bidelta}{\boldsymbol{\delta}}\newcommand{\bivarepsilon}{\boldsymbol{\varepsilon}}\newcommand{\bizeta}{\boldsymbol{\zeta}}\newcommand{\bieta}{\boldsymbol{\eta}}\newcommand{\bitheta}{\boldsymbol{\theta}}\newcommand{\biiota}{\boldsymbol{\iota}}\newcommand{\bikappa}{\boldsymbol{\kappa}}\newcommand{\bilambda}{\boldsymbol{\lambda}}\newcommand{\bimu}{\boldsymbol{\mu}}\newcommand{\binu}{\boldsymbol{\nu}}\newcommand{\bixi}{\boldsymbol{\xi}}\newcommand{\biomicron}{\boldsymbol{\micron}}\newcommand{\bipi}{\boldsymbol{\pi}}\newcommand{\birho}{\boldsymbol{\rho}}\newcommand{\bisigma}{\boldsymbol{\sigma}}\newcommand{\bitau}{\boldsymbol{\tau}}\newcommand{\biupsilon}{\boldsymbol{\upsilon}}\newcommand{\biphi}{\boldsymbol{\phi}}\newcommand{\bichi}{\boldsymbol{\chi}}\newcommand{\bipsi}{\boldsymbol{\psi}}\newcommand{\biomega}{\boldsymbol{\omega}}\begin{equation}\tag{2}d\Phi \left( {r,\theta } \right) = T\left( {r,\theta } \right)\cos \beta \left( {r,\theta } \right)r\,d\theta {\rm }\end{equation}

where *r* is the radius and \newcommand{\bialpha}{\boldsymbol{\alpha}}\newcommand{\bibeta}{\boldsymbol{\beta}}\newcommand{\bigamma}{\boldsymbol{\gamma}}\newcommand{\bidelta}{\boldsymbol{\delta}}\newcommand{\bivarepsilon}{\boldsymbol{\varepsilon}}\newcommand{\bizeta}{\boldsymbol{\zeta}}\newcommand{\bieta}{\boldsymbol{\eta}}\newcommand{\bitheta}{\boldsymbol{\theta}}\newcommand{\biiota}{\boldsymbol{\iota}}\newcommand{\bikappa}{\boldsymbol{\kappa}}\newcommand{\bilambda}{\boldsymbol{\lambda}}\newcommand{\bimu}{\boldsymbol{\mu}}\newcommand{\binu}{\boldsymbol{\nu}}\newcommand{\bixi}{\boldsymbol{\xi}}\newcommand{\biomicron}{\boldsymbol{\micron}}\newcommand{\bipi}{\boldsymbol{\pi}}\newcommand{\birho}{\boldsymbol{\rho}}\newcommand{\bisigma}{\boldsymbol{\sigma}}\newcommand{\bitau}{\boldsymbol{\tau}}\newcommand{\biupsilon}{\boldsymbol{\upsilon}}\newcommand{\biphi}{\boldsymbol{\phi}}\newcommand{\bichi}{\boldsymbol{\chi}}\newcommand{\bipsi}{\boldsymbol{\psi}}\newcommand{\biomega}{\boldsymbol{\omega}}\(\theta \) is the angular position.

This equation is a two-dimensional equivalent of Gauss' flux theorem, where \newcommand{\bialpha}{\boldsymbol{\alpha}}\newcommand{\bibeta}{\boldsymbol{\beta}}\newcommand{\bigamma}{\boldsymbol{\gamma}}\newcommand{\bidelta}{\boldsymbol{\delta}}\newcommand{\bivarepsilon}{\boldsymbol{\varepsilon}}\newcommand{\bizeta}{\boldsymbol{\zeta}}\newcommand{\bieta}{\boldsymbol{\eta}}\newcommand{\bitheta}{\boldsymbol{\theta}}\newcommand{\biiota}{\boldsymbol{\iota}}\newcommand{\bikappa}{\boldsymbol{\kappa}}\newcommand{\bilambda}{\boldsymbol{\lambda}}\newcommand{\bimu}{\boldsymbol{\mu}}\newcommand{\binu}{\boldsymbol{\nu}}\newcommand{\bixi}{\boldsymbol{\xi}}\newcommand{\biomicron}{\boldsymbol{\micron}}\newcommand{\bipi}{\boldsymbol{\pi}}\newcommand{\birho}{\boldsymbol{\rho}}\newcommand{\bisigma}{\boldsymbol{\sigma}}\newcommand{\bitau}{\boldsymbol{\tau}}\newcommand{\biupsilon}{\boldsymbol{\upsilon}}\newcommand{\biphi}{\boldsymbol{\phi}}\newcommand{\bichi}{\boldsymbol{\chi}}\newcommand{\bipsi}{\boldsymbol{\psi}}\newcommand{\biomega}{\boldsymbol{\omega}}\(T\cos \beta\, dl\) is the flux \newcommand{\bialpha}{\boldsymbol{\alpha}}\newcommand{\bibeta}{\boldsymbol{\beta}}\newcommand{\bigamma}{\boldsymbol{\gamma}}\newcommand{\bidelta}{\boldsymbol{\delta}}\newcommand{\bivarepsilon}{\boldsymbol{\varepsilon}}\newcommand{\bizeta}{\boldsymbol{\zeta}}\newcommand{\bieta}{\boldsymbol{\eta}}\newcommand{\bitheta}{\boldsymbol{\theta}}\newcommand{\biiota}{\boldsymbol{\iota}}\newcommand{\bikappa}{\boldsymbol{\kappa}}\newcommand{\bilambda}{\boldsymbol{\lambda}}\newcommand{\bimu}{\boldsymbol{\mu}}\newcommand{\binu}{\boldsymbol{\nu}}\newcommand{\bixi}{\boldsymbol{\xi}}\newcommand{\biomicron}{\boldsymbol{\micron}}\newcommand{\bipi}{\boldsymbol{\pi}}\newcommand{\birho}{\boldsymbol{\rho}}\newcommand{\bisigma}{\boldsymbol{\sigma}}\newcommand{\bitau}{\boldsymbol{\tau}}\newcommand{\biupsilon}{\boldsymbol{\upsilon}}\newcommand{\biphi}{\boldsymbol{\phi}}\newcommand{\bichi}{\boldsymbol{\chi}}\newcommand{\bipsi}{\boldsymbol{\psi}}\newcommand{\biomega}{\boldsymbol{\omega}}\(d{\rm{\Phi }}\). The flux concept is further explained in Appendix A.

**Figure 3:**
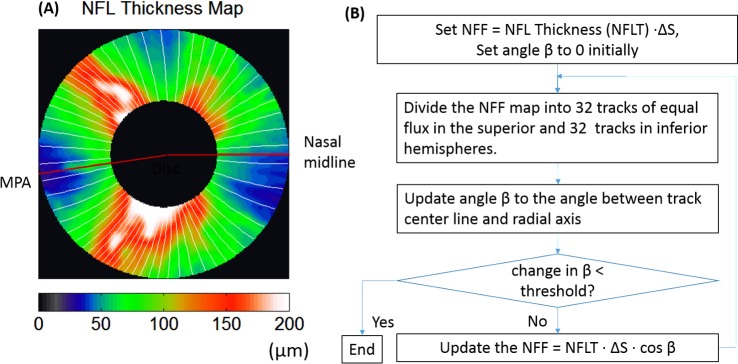
Flux tracing based on the NFL thickness map. (A) For each eye, the NFL thickness map was divided into 64 tracks (white trajectory lines mark the center of each track) composed of 32 tracks each in the superior and inferior hemispheres. In each hemisphere, the tracks were adjusted iteratively by the tracing algorithm so each track had equal flux. The hemispheric division was anchored by the maculopapillary axis and the nasal midline. (B) Flow chart of NFF tracing algorithm. ΔS, the derivative of arc length used to transform thickness to area.

We then divided the total NFL map into tracks of equal flux (Fig. 3A). We defined NFF as the perpendicular cross-sectional area of the nerve fibers. The NFF in each track will be conserved if it follows the course of the nerve fibers. We use this premise to iteratively solve for the nerve fiber trajectory using the OCT-derived NFL thickness map. The iteration started by assuming the angle *β* equaled 0 so that the track centerline positions in each circular NFL profile could be obtained by dividing the total NFF (initially equal to the cross-sectional area) into equal parts (Fig. 3B). This provided an NFL trajectory map *φ_n_*(*r*) that described the angular position of the tracks as a function of radius, *r*, where the subscript *n* was the index of the track. This initial trajectory was then used to estimate the map of cos *β*(*r*, *θ*) in polar coordinates. The map of cos *β* was in turn used to refine the flux density profiles. The refined flux density profiles were then again divided into equal parts to establish a more precise trajectory map. The steps were repeated until *β*(*r*, *θ*) converged as indicated by a change of less than 0.01° between iterations.

We found that this method converged well when the NFF density map was divided into 32 tracks in superior hemisphere and 32 tracks in inferior hemisphere. The hemispheres were divided by the horizontal midline nasal to the disc and by the temporal MPA. The hemispheric division prevents the effects of segmentation and centration errors from propagating across the midline. We further constrained the trajectory of the tracks to arcuate shapes described by the mathematic model^24^

\newcommand{\bialpha}{\boldsymbol{\alpha}}\newcommand{\bibeta}{\boldsymbol{\beta}}\newcommand{\bigamma}{\boldsymbol{\gamma}}\newcommand{\bidelta}{\boldsymbol{\delta}}\newcommand{\bivarepsilon}{\boldsymbol{\varepsilon}}\newcommand{\bizeta}{\boldsymbol{\zeta}}\newcommand{\bieta}{\boldsymbol{\eta}}\newcommand{\bitheta}{\boldsymbol{\theta}}\newcommand{\biiota}{\boldsymbol{\iota}}\newcommand{\bikappa}{\boldsymbol{\kappa}}\newcommand{\bilambda}{\boldsymbol{\lambda}}\newcommand{\bimu}{\boldsymbol{\mu}}\newcommand{\binu}{\boldsymbol{\nu}}\newcommand{\bixi}{\boldsymbol{\xi}}\newcommand{\biomicron}{\boldsymbol{\micron}}\newcommand{\bipi}{\boldsymbol{\pi}}\newcommand{\birho}{\boldsymbol{\rho}}\newcommand{\bisigma}{\boldsymbol{\sigma}}\newcommand{\bitau}{\boldsymbol{\tau}}\newcommand{\biupsilon}{\boldsymbol{\upsilon}}\newcommand{\biphi}{\boldsymbol{\phi}}\newcommand{\bichi}{\boldsymbol{\chi}}\newcommand{\bipsi}{\boldsymbol{\psi}}\newcommand{\biomega}{\boldsymbol{\omega}}\begin{equation}\tag{3}{\varphi _n}\left( r \right) = {a_n} + {b_n}{(r - {r_0})^{{c_n}}}{\rm }\end{equation}

where *a_n_* was the angular position of the track at reference radius *r*_0_ = 0.85 mm, *b_n_* was a real number, and *c_n_* was a positive real number that determined how quickly the arc changed direction with increasing radius.

### Flux Compensation for Ganglion Cells in the Macular Area

In most of the peripapillary region, the number of nerve fibers, and therefore the NFF, is approximately conserved because the ganglion cell density is negligible. However, on the macular side, the ganglion cell concentration rises with proximity to the fovea. This requires compensation as nerve fibers connect to ganglion cell bodies, and the corresponding flux line terminates.

**Figure 4:**
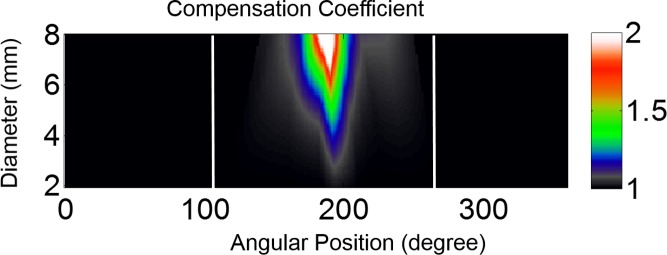
Compensation coefficient in the macular area near the MPA. The annular map is displayed in polar coordinates (center at disc). The angular positions of 0° and 360° corresponded to the nasal midline and the angles increment counterclockwise for the right eye and clockwise for the left eye. Compensation coefficients for angular positions <110° or >270° (two white lines) are manually set to 1. The compensated NFF approximately conserved along the track in wide-field peripapillary scans.

To compensate for the flux reduction along the nerve fiber trajectory in the macular area, we assumed that the reduction of flux was proportional to the integration of ganglion cells along the tracks. Based on a typical ganglion cell density map^25^ and an averaged nerve fiber trajectory,^24^ we obtained a compensation coefficient for the NFL thickness map (Fig. 4). Details of the calculation of the compensation coefficient are presented in Appendix A. The compensation coefficients were only calculated for 110° to 270°, and manually set to 1 elsewhere because we assume that the ganglion cell density in those area is very low. This compensation coefficient was applied in the initial step of flow chart (Fig. 3B), where the initial NFF map = NFL thickness map × \newcommand{\bialpha}{\boldsymbol{\alpha}}\newcommand{\bibeta}{\boldsymbol{\beta}}\newcommand{\bigamma}{\boldsymbol{\gamma}}\newcommand{\bidelta}{\boldsymbol{\delta}}\newcommand{\bivarepsilon}{\boldsymbol{\varepsilon}}\newcommand{\bizeta}{\boldsymbol{\zeta}}\newcommand{\bieta}{\boldsymbol{\eta}}\newcommand{\bitheta}{\boldsymbol{\theta}}\newcommand{\biiota}{\boldsymbol{\iota}}\newcommand{\bikappa}{\boldsymbol{\kappa}}\newcommand{\bilambda}{\boldsymbol{\lambda}}\newcommand{\bimu}{\boldsymbol{\mu}}\newcommand{\binu}{\boldsymbol{\nu}}\newcommand{\bixi}{\boldsymbol{\xi}}\newcommand{\biomicron}{\boldsymbol{\micron}}\newcommand{\bipi}{\boldsymbol{\pi}}\newcommand{\birho}{\boldsymbol{\rho}}\newcommand{\bisigma}{\boldsymbol{\sigma}}\newcommand{\bitau}{\boldsymbol{\tau}}\newcommand{\biupsilon}{\boldsymbol{\upsilon}}\newcommand{\biphi}{\boldsymbol{\phi}}\newcommand{\bichi}{\boldsymbol{\chi}}\newcommand{\bipsi}{\boldsymbol{\psi}}\newcommand{\biomega}{\boldsymbol{\omega}}\(\gamma \) × \newcommand{\bialpha}{\boldsymbol{\alpha}}\newcommand{\bibeta}{\boldsymbol{\beta}}\newcommand{\bigamma}{\boldsymbol{\gamma}}\newcommand{\bidelta}{\boldsymbol{\delta}}\newcommand{\bivarepsilon}{\boldsymbol{\varepsilon}}\newcommand{\bizeta}{\boldsymbol{\zeta}}\newcommand{\bieta}{\boldsymbol{\eta}}\newcommand{\bitheta}{\boldsymbol{\theta}}\newcommand{\biiota}{\boldsymbol{\iota}}\newcommand{\bikappa}{\boldsymbol{\kappa}}\newcommand{\bilambda}{\boldsymbol{\lambda}}\newcommand{\bimu}{\boldsymbol{\mu}}\newcommand{\binu}{\boldsymbol{\nu}}\newcommand{\bixi}{\boldsymbol{\xi}}\newcommand{\biomicron}{\boldsymbol{\micron}}\newcommand{\bipi}{\boldsymbol{\pi}}\newcommand{\birho}{\boldsymbol{\rho}}\newcommand{\bisigma}{\boldsymbol{\sigma}}\newcommand{\bitau}{\boldsymbol{\tau}}\newcommand{\biupsilon}{\boldsymbol{\upsilon}}\newcommand{\biphi}{\boldsymbol{\phi}}\newcommand{\bichi}{\boldsymbol{\chi}}\newcommand{\bipsi}{\boldsymbol{\psi}}\newcommand{\biomega}{\boldsymbol{\omega}}\(\cos \beta \). The compensation was only significant in the outer circles of the temporal side of the peripapillary NFL thickness map (Fig. 4). Notice this compensation was only used to calculate the nerve fiber trajectory from the healthy group. This compensation was not used to calculate the trajectory in glaucoma patients as the ganglion cell density distribution would be affected by glaucoma and cannot be estimated accurately.

### Average Trajectory and Sector Analysis

The average human NFL trajectory map was calculated among normal group. First, trajectory maps were obtained using the iterative method described above. Each trajectory map includes 64 tracks divided by 64 border lines. The border lines were represented by angular position and radii (1.5∼3.5 mm). Equation 3 was applied to obtain a mathematic model for the border lines. Using the mathematic model, we could extend the trajectory to D < 3 mm or D > 3.5 mm. The central position of a track is averaged from its two borders. The trajectory maps of healthy individual eyes were averaged to obtain the normal average trajectory map. The normal average cos *β* map was then derived from the normal average trajectory map.

The normal average trajectory map and the cosine correction map were then applied to each test eye to calculate the NFF-per-track map. First, the NFL thickness map with magnification correction was transferred to the nerve fiber area density map based on the radius. Thus, the cosine corrected nerve fiber area became the NFF. Then the NFF-per-track was the arc integration of the NFF along the circle for each track.

The tracks were further aggregated into eight sectors according to a scheme originally devised by Garway-Heath et al.^26^ and modified by us^27^ to follow nerve fiber trajectories over a wide map. The nerve fiber tracks were grouped into sectors according to the angular demarcation positions defined by Garway-Heath et al.^26^ They defined the sectors at the disc rim (1.7-mm diameter anchor circle). We extrapolated these sector-dividing lines outward to larger radii according to the normal average trajectory map. This allows us to define NFF sectors over extended areas and correlate them with matching VF sectors on the standard Humphrey 24-2 perimetry (see Fig. 8A). The superior and inferior hemispheres were each divided into four sectors: papillomacular (PM), inner arcuate (IA), outer arcuate (OA), and temporal field (TF).

The NFF was summed in each sector or the whole area of the NFF map to calculate the sector NFF and the overall NFF.

## Results

There were 25 participants enrolled in the healthy control group and 10 in the glaucoma group (Table). One healthy eye was excluded because of poor image quality. Among the 10 glaucoma patients, eight had mild glaucoma (MD > −6 dB) and two had moderate glaucoma (MD between −6 and −12 dB) according to the Hodapp-Parrish-Anderson Staging Criteria.^28^

**Table:**
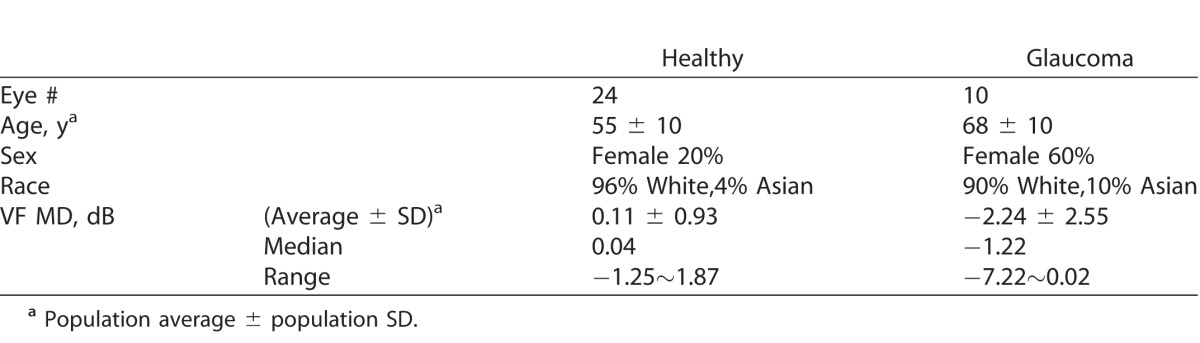
Subject Characteristics

**Figure 5:**
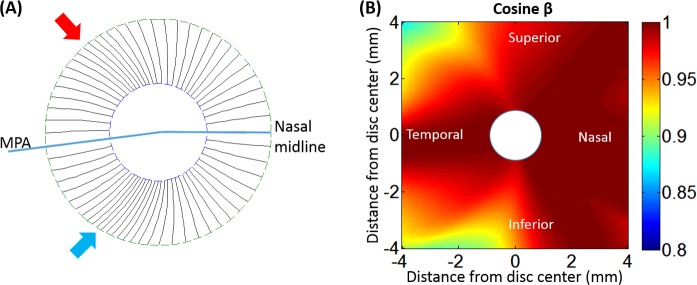
Averaged NFF tracks and NFL cross-section area correction. (A) Nerve fiber track trajectories in the annular region between the diameters of 3 and 7 mm were averaged from 24 healthy subjects. The NFF map was were evenly divided by 64 tracks (black lines mark track boundaries) between the superior and inferior hemispheres. However, the track width is not even. The narrowest tracks were located at red and blue arrow; (B) The cos β map in an 8 × 8-mm area based on the skew of the trajectories.

The nerve fiber tracking algorithm was applied to the 24 healthy eyes. After magnification correction, the trajectories of the nerve fiber tracks were averaged for the healthy group (Fig. 5A). The cos *β* map (Fig. 5B) was then calculated from the angle *β* between the trajectories and radial lines originating from the disc center. The value of cos *β* outside the annular region between the diameters of 3 and 7 mm was calculated based on extrapolation using the mathematic model in Equation 3. Based on the averaged nerve fiber tracks (Fig. 5A), the track widths were not even. The track density was highest in the superior-temporal and inferior-temporal bundles, which correspond to the two humps in the NFL thickness profile. The track density is lowest in the nasal area followed by the temporal area, which corresponds to the areas having the thinnest NFL. As evident in the cos *β* map (Fig. 5B), there is little angle correction of NFL thickness in the nasal area where the trajectories were nearly perfectly radial. The greatest deviation from the radial orientation occurred further from the disc, at diameters >*β*6 mm, in the superior-temporal, inferior, and inferior-temporal nerve fiber tracks.

**Figure 6:**
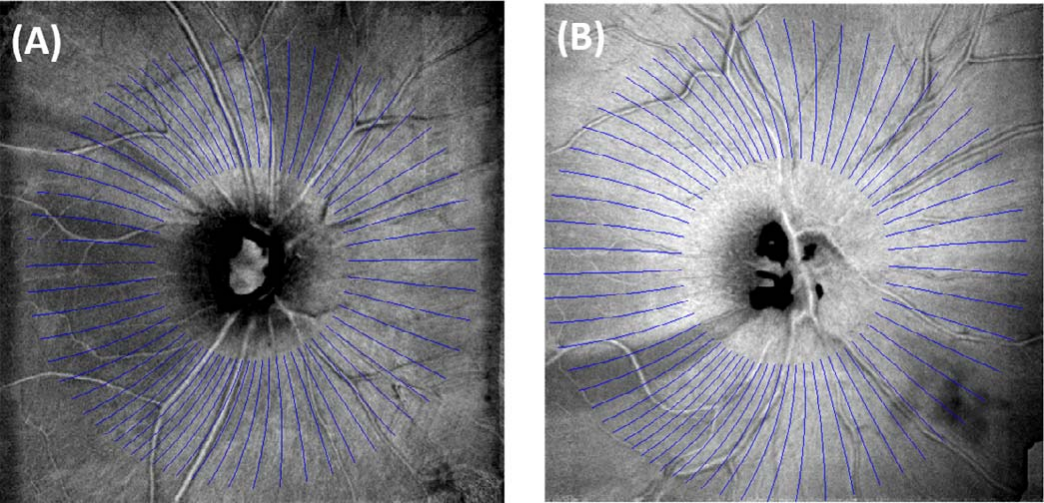
Overlay of NFF track trajectories (blue lines drawn between 3- and 7-mm diameters) on the en face OCT (gray scale image) of the NFL slab in two glaucoma subjects. (A, B) The trajectory map was registered to the en face OCT by the position of the disc and the orientation of the MPA. Magnification was adjusted by the axial eye length.

The NFF track trajectory map obtained from the 24 healthy eyes was superimposed on the en face OCT image of the NFL slab in two glaucomatous eyes with the most well-defined bundle defects (Fig. 6). The bundle defects followed the NFF trajectories well in these two eyes, as well as in other glaucomatous eyes with visible bundle defects. We note that in the nasal region between +60° to −60°, the NFF trajectories are radial, while in the superior and inferior regions the NFF trajectories curve temporally toward the macula.

**Figure 7:**
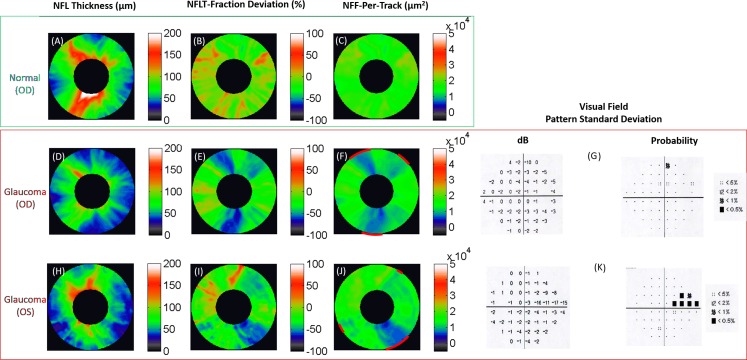
NFL thickness (NFLT), NFLT fraction deviation, and NFF-per-track maps for a healthy (A, B) and two glaucomatous eyes (C–F, H–J). The annular maps spanned diameters between 3 and 7 mm. the fraction deviation map is based on (NFLT map-normal reference)/ normal reference × 100%. Both the NFLT fraction deviation map and NFF-per-track maps in the glaucomatous eyes showed arcuate wedge defects (marked by red arcs) that followed the tracks. Both inferior NFF defects showed subtle raised patterns (green relief within the blue wedge depressions) consistent with major blood vessels. The VF PSD of two glaucomatous eyes were also showed in (G) and (K), including both dB and probability plots. dB, decibel.

The NFL thickness map of all healthy eyes showed the thick superior and inferior arcuate bundles (double hump pattern) (Fig. 7A), whereas the NFF-per-track map was approximately uniform (Fig. 7B). The NFL thickness map of glaucoma subjects showed loss of the normal double-hump pattern (Figs. 7D, 7H). The NFF-per-track map showed arcuate pattern of nerve fiber loss in the superior or inferior regions (Figs. 7F, 7J), which corresponded with arcuate or nasal VF defects. The pattern is similar to the NFL thickness fraction deviation map (Figs. 7E, 7I). The nerve fiber defect generally followed the arcuate course of the flux tracks well and was relatively uniform in depth along the course of the tracks. This arcuate wedge shaped anatomic pattern of loss helped confirm that the defects were real and not artifacts. Blood vessels within the wedge defects were visible in some cases as subtle relief within the depressions. The particular examples shown in Figure 7 were chosen to demonstrate the typical patterns of biarcuate and inferior arcuate defects. They suggest that nerve fiber defects are easier to recognize on the NFF maps than traditional NFL thickness maps, and comparable to NFL thickness fraction deviation map.

**Figure 8:**
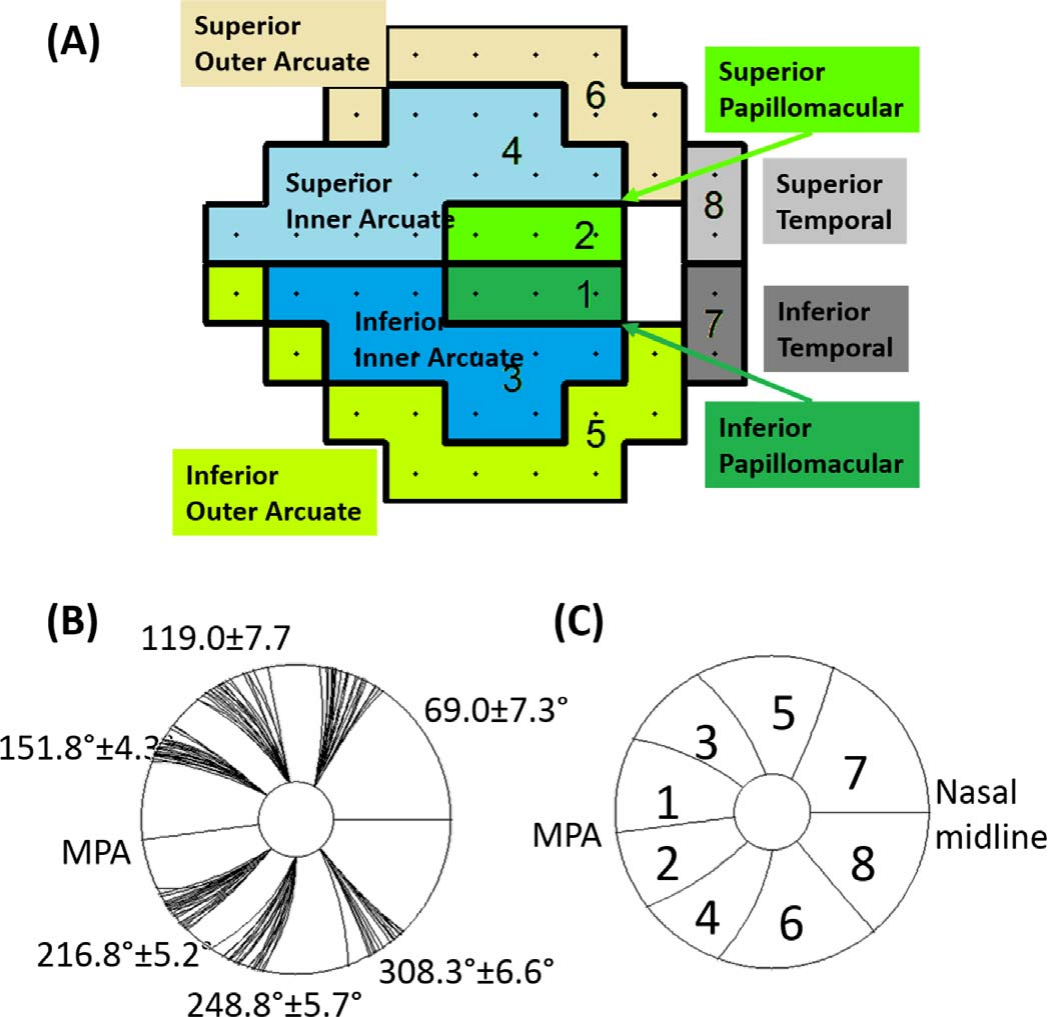
NFF maps were divided into eight sectors using the Garway-Heath et al.^26^ scheme to correlate VF and the NFL thickness. The scheme started at a small circle with a 1.7-mm diameter and then extended to the NFL at greater diameters (up to 7 mm) using our results on flux trajectory. (A) The Humphrey 24-2 VF divided into eight sectors. Each hemisphere has four sectors: PM, IA, OA, and TF; (B) after registration, the eight-sector NFF dividing lines in 24 healthy subjects were plotted individually. The average and SD of angular positions of the dividing lines at a radius of 3.5 mm are shown. (C) The eight-sector dividing lines were averaged in the normal group.

The nerve fiber tracks were grouped into sectors according to the angular demarcation positions defined by Garway-Heath et al.^26^ at the disc rim (1.7-mm diameter anchor circle), and the sectors were propagated to larger radii according to the trajectories obtained from the 24 healthy subjects. This sector division was developed to correspond to VF regions on the standard Humphrey 24-2 perimetry (Fig. 8A). The track trajectories at the boundary of these sectors were plotted individually for the 24 healthy subjects (Fig. 8B). The variability of the angular position of the sector boundaries increased with radial distance from the anchor circle. At the 7-mm diameter, the variability ranged between 5° and 8°. Before registration, the MPA angle of the 24 healthy participants was 6.8° ± 3.1° (mean ± standard deviation [SD]). Using data from the healthy group, we extended the Garway-Heath et al.^26^ sectors from the original definition at nerve head to the annular region between 1.7 and 7-mm diameter (Fig. 8C). Together with the normative cos *β* map (Fig. 5B), we performed NFF analysis on a test eye based on the NFL trajectory and VF regional correspondence of the average eye.

**Figure 9:**
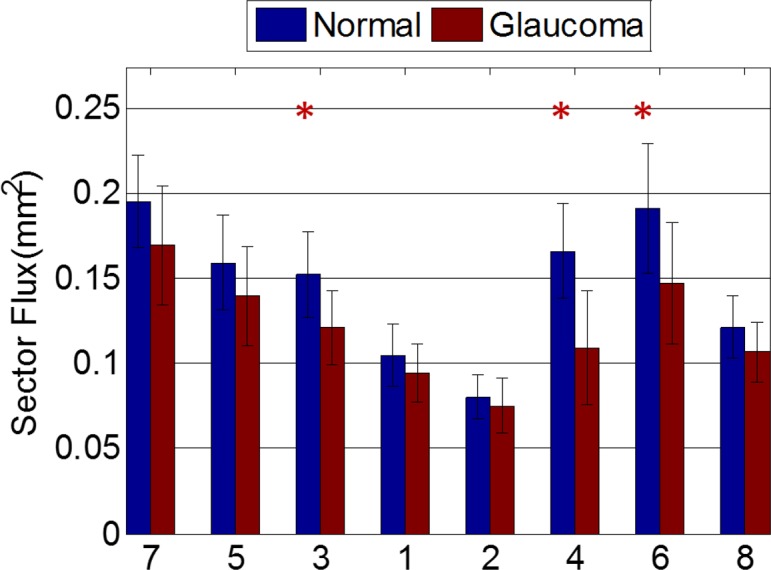
Sectoral NFF distribution in normal and glaucoma group. *, sector with significant difference (P < 0.05/8 after Bonferroni correction) between the healthy and glaucoma groups. Error bars: SD.

**Figure 10:**
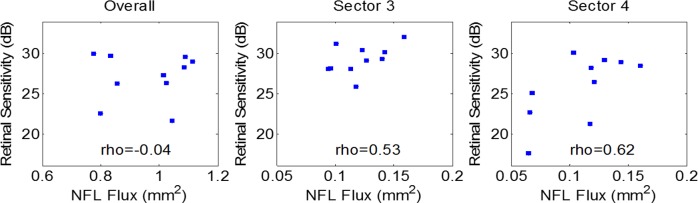
Correlation between NFF and VF retinal sensitivity in glaucoma patients. The flux value was the summation of the NFF in whole area or specific sectors, while the VF value was the sensitivity (dB) averaged in the whole area or specific sectors. We chose sectors three and four because they corresponded to the inferior and superior arcuate sectors in VF. Rho is the Spearman correlation coefficient.

Sector analysis of NFF showed that the glaucoma group had reduced flux in sectors three (superior-temporal), four (inferior-temporal), and six (inferior) (*P* < 0.001 for each sector using two-samples *t*-test, Fig. 9). We counted the percentage of abnormal sectors in the glaucoma group using a 5% cutoff from the normal reference. In sectors three, four, and six, 40% to 60% of eyes in glaucoma group had abnormally low flux at the cutoff value. The abnormality rates of other sectors were between 10% and 20%. The Spearman correlation between VF retinal sensitivity and NFF were highest (ρ ≥ 0.53) in sectors three and four (Fig. 10), which also had the most glaucoma damage in both hemispheres. The ρ value in the other sectors ranged between 0.0 and 0.37. In contrast, the overall NFF and the overall VF sensitivity were poorly correlated.

## Discussion

It was 21-years ago that OCT scan speed, at 40 Hz, was more than 2000 times slower than the system used in this study. For that reason, the original glaucoma scan pattern used a fixed 3.4-mm diameter circular scan to image a cylindrical section of the NFL that efficiently transected all of the retinal nerve fibers.^1,29^ With the 70- to 100-kHz speeds of current commercial ophthalmic OCT systems, three-dimensional volumetric scanning of larger areas can be easily accomplished. Despite technologic advances, NFL diagnostic analysis has remained stuck with the enshrined 3.4-mm diameter cylindric section. The purpose of this study was to develop a method to break with tradition and fully use the anatomic information in a large volumetric scan of the peripapillary NFL. However, as the NFL is traced from this small circle outward, the nerve fibers do not traverse straight lines, but instead, they become increasingly skewed as they course away from the disc and arc around the fovea. Consequently, glaucomatous nerve fiber bundle defects appear in different places at different circle sampling diameters. The arcuate course must be known to analyze NFL bundle defects over a wide range of sampling diameters. A second complication, which had not been recognized before, is that the significantly skewed angles in the superior-temporal and inferior-temporal bundles lead to significant apparent increases in the NFL cross-sectional area due to the oblique sampling angle.

To solve these problems, we applied a cosine correction to the NFL thickness to recover the perpendicular cross-section. The total perpendicular cross-section of the NFL should be approximately conserved over a range of sampling circle diameters, as it represents all the nerve fibers entering the optic nerve. The approximation is valid if we make the reasonable assumption that each nerve fiber and associated glial support maintains an approximately constant perpendicular cross-sectional area as they traverse the peripapillary retina. Further we assumed that the number of fiber originations on the RGC in most peripapillary areas is negligible compared with the total number of RGCs, with the exception of the temporal quadrant. The temporal quadrant extends into the macula, which has a high concentration of RGCs. Therefore in the temporal quadrant we compensated for the expected flux change according to the known RGC distribution in humans.^25^ By these methods, we were able to trace the flux distribution over an extended range of peripapillary circular radii and recover the trajectory of nerve fibers using information inherent in the NFL thickness map. We recognized that our method of linking NFL thickness to nerve fiber trajectory is analogous to the way Gauss linked electric field flux to electric charges. Our mathematic equations are borrowed from Gauss flux theorem, the derivations of which are in Appendix A.

**Figure 11:**
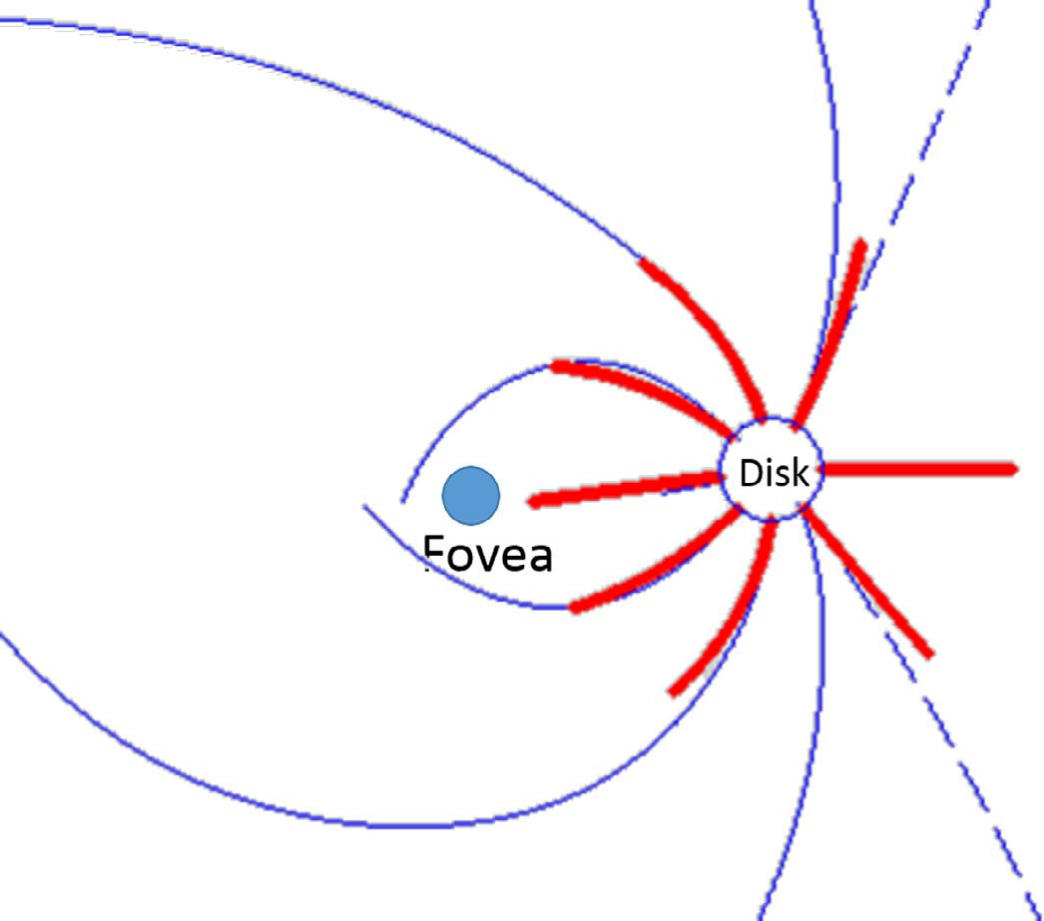
Comparing nerve fiber bundle trajectories between Jansonius' method^24^ (solid line), Jansonius' revised method^30^ (dash lines) based on photographic analysis, and our flux analysis method (thick solid lines). The lines were chosen to match the sector divisions in Garway-Heath et al. method.^26^

We obtained the peripapillary nerve fiber trajectory map from each healthy human subject and then averaged the maps. Our average trajectory map mostly agrees well with what Jansonius et al.^24^ obtained in 2009 by tracing nerve fiber bundles in the temporal, superior, and inferior to the disc from photographs of subjects without diseases affecting RNFL visibility (Fig. 11). An exception is that in the 2009 map of Jansonius et al.^24^, the dividing lines for sectors 5/7 and 6/8 are more temporal than what we found. However, these dividing lines do agree with a later 2012 study by Jansonius et al.,^30^ that traced nerve fiber bundle trajectory in the nasal retina (−60° to approximately 60°). Jansonius et al.^30^ pointed out that there is a “singularity” area near 60° and −60°, where the nerve fibers are highly divergent and the mathematic model extrapolate out to a region with no nerve fibers and where the trajectory is poorly defined. We did find moderately larger population variation in the trajectory in the superior and inferior regions (see sector 3/5, 4/6, 5/7, and 6/8 sector boundary variation in Fig. 8B) where the nerve fibers are more divergent (transitioning from radial to arcuate trajectory). However, we did not find any location with extreme divergence or variability. We believe this is dues to our smaller analytic area (7-mm diameter), which does not contain the extreme divergence found further from the disc in Jansonius' model^24,30^ fit over a larger area (14-mm diameter).

Another method of obtaining the nerve fiber trajectory map has been devised using polarization-sensitive OCT (PS-OCT). Sugita et al.^31^ applied PS-OCT to both the macular and peripapillary areas. In the macular area, where the relatively thin NFL formed distinct bundles, they detected the trajectory by thickness gradient analysis. In the peripapillary area, they used information on the axis of birefringent retardation, which is aligned with the form birefringence of the nerve fibers. Sugita et al.^31^ provided trajectory maps from each healthy human individual, so quantitative comparison is not possible. Qualitatively, their trajectory pattern is similar to our results, showing arcuate courses in the superior and inferior quadrants, and a temporal trajectory that followed the maculopapillary axis. The methods of Sugita et al.^31^ are difficult to apply to a large clinical study because PS-OCT is not commercially available. Our methods could be more easily applied to commercially available Fourier-domain OCT systems, which are generally capable of volumetric scans or multicircular scans of the peripapillary area.

Once the average trajectory and flux maps were obtained from the healthy group, we developed a system to detect deviations from normal in the glaucoma group. The scheme summarized the normal maps into a template of 64 tracks and eight sectors. The NFL map from the test eye is registered with the template at the center of the disc. The NFL cross-sectional area within each track is then translated to NFF by applying the cosine correction using the normal average trajectory map. The NFF within each track and sector can then be statistically compared with the healthy population. By averaging the NFF in each track, the analysis of focal nerve fiber loss can be reduced to a one-dimensional plot of NFF (cross-sectional area per track) versus track angular position, while making use of full information from a wide volumetric OCT scan in the peripapillary region. The advantage of this approach requires further validation in a larger clinical study that uses wide-field scans of the peripapillary NFL.

We also aggregated the NFF tracks into sectors that were designed to correlate with VF regions. Because the NFF sector boundaries follow nerve fiber trajectories, the correspondence with VF sectors should be better than NFL thickness averages based on pie-shaped sectors with straight boundaries. While we do not have a large enough sample size in this study to directly demonstrate this advantage, we were able to demonstrate good correlation of sector NFF with VF sensitivity in the two arcuate sectors (i.e., 3 and 4) that are most commonly and severely damaged by glaucoma. Our sector division is an extension of the method by Garway-Heath et al.^26^ of dividing the disc rim into sectors that correlated with regional VF defects in glaucoma patients. We and other investigators have previously extended the Garway-Heath et al.^26^ system to the peripapillary NFL thickness profile and found good correlation with VF loss in glaucoma patients, especially in sectors three and four.^27,32,33^ Other systems have also been devised to divide the NFL thickness profile into sectors that correlate with VF regions.^34,35^ In all of these studies, NFL thickness correlated with VF better as sector rather than global averages, as we have also found here. Our method of NFF sector analysis makes full use of wide-field volumetric OCT scans and may further enhance VF correlation. This theoretic advantage remains to be confirmed using a larger sample of human subjects.

Our method is limited by the assumption that the nerve fiber trajectory and distribution among healthy human subjects are similar. We followed the Garway-Heath et al.^26^ definition to match the NFF sectors to the VF regions. This is based on the assumption that the subjects in this study all have similar nerve fiber trajectories as the subjects from Garway-Heath et al.,^26^ at least on average. We also imposed the average normal nerve fiber trajectory map in the analysis of glaucoma subjects, which again assumes that glaucoma subjects retain the normal nerve fiber trajectory pattern. Applications of our normal NFF track and sector template to future studies will also rely on this assumption. While it is reasonable to assume that all human eyes have similar anatomy, small variations were observed within our healthy group. Extrapolation of the trajectory map beyond our measurement area of 7-mm diameter is not recommended because any deviation of individual eye from the population average trajectory error would be amplified further from the disc, especially near the borders of sectors 5/7 and 6/8 (+60° and −60° positions), where Jansonius et al.^30^ had found the nerve fiber bundle trajectories to be divergent and more widely variable between individuals. Some of these variations were accounted for by orientation alignment with the MPA and magnification adjustment based on axial eye length. It is possible that further adjustments in the normative NFF templates could be made based on vessel patterns and demographic variables.

We did not attempt to apply our flux theoretic model to directly derive nerve fiber trajectory maps in glaucoma patients because we did not believe such attempts would be valid. Glaucomatous eyes lose nerve fibers while retaining variable amounts of residual glial tissue. Thus, our model assumption that the average perpendicular cross-sectional area (flux) associated with each nerve fiber and supporting glial tissue would be conserved is not valid in a diseased eye. Furthermore, our compensation for nerve fiber origination from macular RGC on the temporal side is based on the known normal RGC distribution, which would not match that of a diseased eye. These model limitations necessitated our two-stage approach of first measuring the normal NFF maps and then applying the template to diseased eyes.

## Conclusions

In summary, we present a novel nerve fiber tracking algorithm using the peripapillary NFL thickness map. Based on the average nerve fiber trajectory map in a group of healthy subjects, we devised a system to calculate the NFF map and quantify it by track and sector in any test subject. The NFF represents the perpendicular cross-sectional area of the nerve fibers and glial tissue and should be better conserved over a wider range of radial distances from the disc compared with raw NFL thickness. The NFF tracks and sector precisely follow the average nerve fiber trajectory over a wide area on peripapillary OCT scans and may allow more sensitive detection of focal damage and more accurate quantification of regional loss. These theoretic advantages need to be tested in a larger study.

## Appendix 1: Flux Theorem

Gauss's flux theorem, originally applied to electric field, can be written as

\newcommand{\bialpha}{\boldsymbol{\alpha}}\newcommand{\bibeta}{\boldsymbol{\beta}}\newcommand{\bigamma}{\boldsymbol{\gamma}}\newcommand{\bidelta}{\boldsymbol{\delta}}\newcommand{\bivarepsilon}{\boldsymbol{\varepsilon}}\newcommand{\bizeta}{\boldsymbol{\zeta}}\newcommand{\bieta}{\boldsymbol{\eta}}\newcommand{\bitheta}{\boldsymbol{\theta}}\newcommand{\biiota}{\boldsymbol{\iota}}\newcommand{\bikappa}{\boldsymbol{\kappa}}\newcommand{\bilambda}{\boldsymbol{\lambda}}\newcommand{\bimu}{\boldsymbol{\mu}}\newcommand{\binu}{\boldsymbol{\nu}}\newcommand{\bixi}{\boldsymbol{\xi}}\newcommand{\biomicron}{\boldsymbol{\micron}}\newcommand{\bipi}{\boldsymbol{\pi}}\newcommand{\birho}{\boldsymbol{\rho}}\newcommand{\bisigma}{\boldsymbol{\sigma}}\newcommand{\bitau}{\boldsymbol{\tau}}\newcommand{\biupsilon}{\boldsymbol{\upsilon}}\newcommand{\biphi}{\boldsymbol{\phi}}\newcommand{\bichi}{\boldsymbol{\chi}}\newcommand{\bipsi}{\boldsymbol{\psi}}\newcommand{\biomega}{\boldsymbol{\omega}}\begin{equation}\tag{A1}Q = {{\int\!\!\!\!\int}\mkern-22mu \bigcirc}_{ s} \varepsilon \vec E \cdot d \vec A {\rm }\end{equation}

where

\newcommand{\bialpha}{\boldsymbol{\alpha}}\newcommand{\bibeta}{\boldsymbol{\beta}}\newcommand{\bigamma}{\boldsymbol{\gamma}}\newcommand{\bidelta}{\boldsymbol{\delta}}\newcommand{\bivarepsilon}{\boldsymbol{\varepsilon}}\newcommand{\bizeta}{\boldsymbol{\zeta}}\newcommand{\bieta}{\boldsymbol{\eta}}\newcommand{\bitheta}{\boldsymbol{\theta}}\newcommand{\biiota}{\boldsymbol{\iota}}\newcommand{\bikappa}{\boldsymbol{\kappa}}\newcommand{\bilambda}{\boldsymbol{\lambda}}\newcommand{\bimu}{\boldsymbol{\mu}}\newcommand{\binu}{\boldsymbol{\nu}}\newcommand{\bixi}{\boldsymbol{\xi}}\newcommand{\biomicron}{\boldsymbol{\micron}}\newcommand{\bipi}{\boldsymbol{\pi}}\newcommand{\birho}{\boldsymbol{\rho}}\newcommand{\bisigma}{\boldsymbol{\sigma}}\newcommand{\bitau}{\boldsymbol{\tau}}\newcommand{\biupsilon}{\boldsymbol{\upsilon}}\newcommand{\biphi}{\boldsymbol{\phi}}\newcommand{\bichi}{\boldsymbol{\chi}}\newcommand{\bipsi}{\boldsymbol{\psi}}\newcommand{\biomega}{\boldsymbol{\omega}}\(Q\) is the total electric charge enclosed in a closed surface S
\newcommand{\bialpha}{\boldsymbol{\alpha}}\newcommand{\bibeta}{\boldsymbol{\beta}}\newcommand{\bigamma}{\boldsymbol{\gamma}}\newcommand{\bidelta}{\boldsymbol{\delta}}\newcommand{\bivarepsilon}{\boldsymbol{\varepsilon}}\newcommand{\bizeta}{\boldsymbol{\zeta}}\newcommand{\bieta}{\boldsymbol{\eta}}\newcommand{\bitheta}{\boldsymbol{\theta}}\newcommand{\biiota}{\boldsymbol{\iota}}\newcommand{\bikappa}{\boldsymbol{\kappa}}\newcommand{\bilambda}{\boldsymbol{\lambda}}\newcommand{\bimu}{\boldsymbol{\mu}}\newcommand{\binu}{\boldsymbol{\nu}}\newcommand{\bixi}{\boldsymbol{\xi}}\newcommand{\biomicron}{\boldsymbol{\micron}}\newcommand{\bipi}{\boldsymbol{\pi}}\newcommand{\birho}{\boldsymbol{\rho}}\newcommand{\bisigma}{\boldsymbol{\sigma}}\newcommand{\bitau}{\boldsymbol{\tau}}\newcommand{\biupsilon}{\boldsymbol{\upsilon}}\newcommand{\biphi}{\boldsymbol{\phi}}\newcommand{\bichi}{\boldsymbol{\chi}}\newcommand{\bipsi}{\boldsymbol{\psi}}\newcommand{\biomega}{\boldsymbol{\omega}}\(\varepsilon \) is the electric permittivity constant
\newcommand{\bialpha}{\boldsymbol{\alpha}}\newcommand{\bibeta}{\boldsymbol{\beta}}\newcommand{\bigamma}{\boldsymbol{\gamma}}\newcommand{\bidelta}{\boldsymbol{\delta}}\newcommand{\bivarepsilon}{\boldsymbol{\varepsilon}}\newcommand{\bizeta}{\boldsymbol{\zeta}}\newcommand{\bieta}{\boldsymbol{\eta}}\newcommand{\bitheta}{\boldsymbol{\theta}}\newcommand{\biiota}{\boldsymbol{\iota}}\newcommand{\bikappa}{\boldsymbol{\kappa}}\newcommand{\bilambda}{\boldsymbol{\lambda}}\newcommand{\bimu}{\boldsymbol{\mu}}\newcommand{\binu}{\boldsymbol{\nu}}\newcommand{\bixi}{\boldsymbol{\xi}}\newcommand{\biomicron}{\boldsymbol{\micron}}\newcommand{\bipi}{\boldsymbol{\pi}}\newcommand{\birho}{\boldsymbol{\rho}}\newcommand{\bisigma}{\boldsymbol{\sigma}}\newcommand{\bitau}{\boldsymbol{\tau}}\newcommand{\biupsilon}{\boldsymbol{\upsilon}}\newcommand{\biphi}{\boldsymbol{\phi}}\newcommand{\bichi}{\boldsymbol{\chi}}\newcommand{\bipsi}{\boldsymbol{\psi}}\newcommand{\biomega}{\boldsymbol{\omega}}\({\vec E} \) is the electric field
\newcommand{\bialpha}{\boldsymbol{\alpha}}\newcommand{\bibeta}{\boldsymbol{\beta}}\newcommand{\bigamma}{\boldsymbol{\gamma}}\newcommand{\bidelta}{\boldsymbol{\delta}}\newcommand{\bivarepsilon}{\boldsymbol{\varepsilon}}\newcommand{\bizeta}{\boldsymbol{\zeta}}\newcommand{\bieta}{\boldsymbol{\eta}}\newcommand{\bitheta}{\boldsymbol{\theta}}\newcommand{\biiota}{\boldsymbol{\iota}}\newcommand{\bikappa}{\boldsymbol{\kappa}}\newcommand{\bilambda}{\boldsymbol{\lambda}}\newcommand{\bimu}{\boldsymbol{\mu}}\newcommand{\binu}{\boldsymbol{\nu}}\newcommand{\bixi}{\boldsymbol{\xi}}\newcommand{\biomicron}{\boldsymbol{\micron}}\newcommand{\bipi}{\boldsymbol{\pi}}\newcommand{\birho}{\boldsymbol{\rho}}\newcommand{\bisigma}{\boldsymbol{\sigma}}\newcommand{\bitau}{\boldsymbol{\tau}}\newcommand{\biupsilon}{\boldsymbol{\upsilon}}\newcommand{\biphi}{\boldsymbol{\phi}}\newcommand{\bichi}{\boldsymbol{\chi}}\newcommand{\bipsi}{\boldsymbol{\psi}}\newcommand{\biomega}{\boldsymbol{\omega}}\({d \vec A} \) is the differential surface element of the enclosure

Electric flux is proportional to the number of electric field lines going through a surface. For a nonuniform electric field, the electric flux \newcommand{\bialpha}{\boldsymbol{\alpha}}\newcommand{\bibeta}{\boldsymbol{\beta}}\newcommand{\bigamma}{\boldsymbol{\gamma}}\newcommand{\bidelta}{\boldsymbol{\delta}}\newcommand{\bivarepsilon}{\boldsymbol{\varepsilon}}\newcommand{\bizeta}{\boldsymbol{\zeta}}\newcommand{\bieta}{\boldsymbol{\eta}}\newcommand{\bitheta}{\boldsymbol{\theta}}\newcommand{\biiota}{\boldsymbol{\iota}}\newcommand{\bikappa}{\boldsymbol{\kappa}}\newcommand{\bilambda}{\boldsymbol{\lambda}}\newcommand{\bimu}{\boldsymbol{\mu}}\newcommand{\binu}{\boldsymbol{\nu}}\newcommand{\bixi}{\boldsymbol{\xi}}\newcommand{\biomicron}{\boldsymbol{\micron}}\newcommand{\bipi}{\boldsymbol{\pi}}\newcommand{\birho}{\boldsymbol{\rho}}\newcommand{\bisigma}{\boldsymbol{\sigma}}\newcommand{\bitau}{\boldsymbol{\tau}}\newcommand{\biupsilon}{\boldsymbol{\upsilon}}\newcommand{\biphi}{\boldsymbol{\phi}}\newcommand{\bichi}{\boldsymbol{\chi}}\newcommand{\bipsi}{\boldsymbol{\psi}}\newcommand{\biomega}{\boldsymbol{\omega}}\(d{\Phi _E}\) through a small surface area \newcommand{\bialpha}{\boldsymbol{\alpha}}\newcommand{\bibeta}{\boldsymbol{\beta}}\newcommand{\bigamma}{\boldsymbol{\gamma}}\newcommand{\bidelta}{\boldsymbol{\delta}}\newcommand{\bivarepsilon}{\boldsymbol{\varepsilon}}\newcommand{\bizeta}{\boldsymbol{\zeta}}\newcommand{\bieta}{\boldsymbol{\eta}}\newcommand{\bitheta}{\boldsymbol{\theta}}\newcommand{\biiota}{\boldsymbol{\iota}}\newcommand{\bikappa}{\boldsymbol{\kappa}}\newcommand{\bilambda}{\boldsymbol{\lambda}}\newcommand{\bimu}{\boldsymbol{\mu}}\newcommand{\binu}{\boldsymbol{\nu}}\newcommand{\bixi}{\boldsymbol{\xi}}\newcommand{\biomicron}{\boldsymbol{\micron}}\newcommand{\bipi}{\boldsymbol{\pi}}\newcommand{\birho}{\boldsymbol{\rho}}\newcommand{\bisigma}{\boldsymbol{\sigma}}\newcommand{\bitau}{\boldsymbol{\tau}}\newcommand{\biupsilon}{\boldsymbol{\upsilon}}\newcommand{\biphi}{\boldsymbol{\phi}}\newcommand{\bichi}{\boldsymbol{\chi}}\newcommand{\bipsi}{\boldsymbol{\psi}}\newcommand{\biomega}{\boldsymbol{\omega}}\(d\vec A \) is given by

\newcommand{\bialpha}{\boldsymbol{\alpha}}\newcommand{\bibeta}{\boldsymbol{\beta}}\newcommand{\bigamma}{\boldsymbol{\gamma}}\newcommand{\bidelta}{\boldsymbol{\delta}}\newcommand{\bivarepsilon}{\boldsymbol{\varepsilon}}\newcommand{\bizeta}{\boldsymbol{\zeta}}\newcommand{\bieta}{\boldsymbol{\eta}}\newcommand{\bitheta}{\boldsymbol{\theta}}\newcommand{\biiota}{\boldsymbol{\iota}}\newcommand{\bikappa}{\boldsymbol{\kappa}}\newcommand{\bilambda}{\boldsymbol{\lambda}}\newcommand{\bimu}{\boldsymbol{\mu}}\newcommand{\binu}{\boldsymbol{\nu}}\newcommand{\bixi}{\boldsymbol{\xi}}\newcommand{\biomicron}{\boldsymbol{\micron}}\newcommand{\bipi}{\boldsymbol{\pi}}\newcommand{\birho}{\boldsymbol{\rho}}\newcommand{\bisigma}{\boldsymbol{\sigma}}\newcommand{\bitau}{\boldsymbol{\tau}}\newcommand{\biupsilon}{\boldsymbol{\upsilon}}\newcommand{\biphi}{\boldsymbol{\phi}}\newcommand{\bichi}{\boldsymbol{\chi}}\newcommand{\bipsi}{\boldsymbol{\psi}}\newcommand{\biomega}{\boldsymbol{\omega}}\begin{equation}\tag{A2}d{\Phi _E} = \vec E \cdot d \vec A {\rm }\end{equation}

The following NFF theorem makes clear how the NFF is analogous to an electric flux. First, we calculate the cross-section area of the retinal nerve fiber layer enclosed in a sampling circle C,

\newcommand{\bialpha}{\boldsymbol{\alpha}}\newcommand{\bibeta}{\boldsymbol{\beta}}\newcommand{\bigamma}{\boldsymbol{\gamma}}\newcommand{\bidelta}{\boldsymbol{\delta}}\newcommand{\bivarepsilon}{\boldsymbol{\varepsilon}}\newcommand{\bizeta}{\boldsymbol{\zeta}}\newcommand{\bieta}{\boldsymbol{\eta}}\newcommand{\bitheta}{\boldsymbol{\theta}}\newcommand{\biiota}{\boldsymbol{\iota}}\newcommand{\bikappa}{\boldsymbol{\kappa}}\newcommand{\bilambda}{\boldsymbol{\lambda}}\newcommand{\bimu}{\boldsymbol{\mu}}\newcommand{\binu}{\boldsymbol{\nu}}\newcommand{\bixi}{\boldsymbol{\xi}}\newcommand{\biomicron}{\boldsymbol{\micron}}\newcommand{\bipi}{\boldsymbol{\pi}}\newcommand{\birho}{\boldsymbol{\rho}}\newcommand{\bisigma}{\boldsymbol{\sigma}}\newcommand{\bitau}{\boldsymbol{\tau}}\newcommand{\biupsilon}{\boldsymbol{\upsilon}}\newcommand{\biphi}{\boldsymbol{\phi}}\newcommand{\bichi}{\boldsymbol{\chi}}\newcommand{\bipsi}{\boldsymbol{\psi}}\newcommand{\biomega}{\boldsymbol{\omega}}\begin{equation}\tag{A3}{A_{NFL}} = \oint_c a\ \vec N \cdot d \vec l {\rm }\end{equation}

where

\newcommand{\bialpha}{\boldsymbol{\alpha}}\newcommand{\bibeta}{\boldsymbol{\beta}}\newcommand{\bigamma}{\boldsymbol{\gamma}}\newcommand{\bidelta}{\boldsymbol{\delta}}\newcommand{\bivarepsilon}{\boldsymbol{\varepsilon}}\newcommand{\bizeta}{\boldsymbol{\zeta}}\newcommand{\bieta}{\boldsymbol{\eta}}\newcommand{\bitheta}{\boldsymbol{\theta}}\newcommand{\biiota}{\boldsymbol{\iota}}\newcommand{\bikappa}{\boldsymbol{\kappa}}\newcommand{\bilambda}{\boldsymbol{\lambda}}\newcommand{\bimu}{\boldsymbol{\mu}}\newcommand{\binu}{\boldsymbol{\nu}}\newcommand{\bixi}{\boldsymbol{\xi}}\newcommand{\biomicron}{\boldsymbol{\micron}}\newcommand{\bipi}{\boldsymbol{\pi}}\newcommand{\birho}{\boldsymbol{\rho}}\newcommand{\bisigma}{\boldsymbol{\sigma}}\newcommand{\bitau}{\boldsymbol{\tau}}\newcommand{\biupsilon}{\boldsymbol{\upsilon}}\newcommand{\biphi}{\boldsymbol{\phi}}\newcommand{\bichi}{\boldsymbol{\chi}}\newcommand{\bipsi}{\boldsymbol{\psi}}\newcommand{\biomega}{\boldsymbol{\omega}}\({A_{NFL}}\) is the total cross-section area of the retinal nerve fibers enclosed in a sampling circle C
\newcommand{\bialpha}{\boldsymbol{\alpha}}\newcommand{\bibeta}{\boldsymbol{\beta}}\newcommand{\bigamma}{\boldsymbol{\gamma}}\newcommand{\bidelta}{\boldsymbol{\delta}}\newcommand{\bivarepsilon}{\boldsymbol{\varepsilon}}\newcommand{\bizeta}{\boldsymbol{\zeta}}\newcommand{\bieta}{\boldsymbol{\eta}}\newcommand{\bitheta}{\boldsymbol{\theta}}\newcommand{\biiota}{\boldsymbol{\iota}}\newcommand{\bikappa}{\boldsymbol{\kappa}}\newcommand{\bilambda}{\boldsymbol{\lambda}}\newcommand{\bimu}{\boldsymbol{\mu}}\newcommand{\binu}{\boldsymbol{\nu}}\newcommand{\bixi}{\boldsymbol{\xi}}\newcommand{\biomicron}{\boldsymbol{\micron}}\newcommand{\bipi}{\boldsymbol{\pi}}\newcommand{\birho}{\boldsymbol{\rho}}\newcommand{\bisigma}{\boldsymbol{\sigma}}\newcommand{\bitau}{\boldsymbol{\tau}}\newcommand{\biupsilon}{\boldsymbol{\upsilon}}\newcommand{\biphi}{\boldsymbol{\phi}}\newcommand{\bichi}{\boldsymbol{\chi}}\newcommand{\bipsi}{\boldsymbol{\psi}}\newcommand{\biomega}{\boldsymbol{\omega}}\(a\) is the average perpendicular cross-section area of single nerve fiber and its associated glial support tissue
\newcommand{\bialpha}{\boldsymbol{\alpha}}\newcommand{\bibeta}{\boldsymbol{\beta}}\newcommand{\bigamma}{\boldsymbol{\gamma}}\newcommand{\bidelta}{\boldsymbol{\delta}}\newcommand{\bivarepsilon}{\boldsymbol{\varepsilon}}\newcommand{\bizeta}{\boldsymbol{\zeta}}\newcommand{\bieta}{\boldsymbol{\eta}}\newcommand{\bitheta}{\boldsymbol{\theta}}\newcommand{\biiota}{\boldsymbol{\iota}}\newcommand{\bikappa}{\boldsymbol{\kappa}}\newcommand{\bilambda}{\boldsymbol{\lambda}}\newcommand{\bimu}{\boldsymbol{\mu}}\newcommand{\binu}{\boldsymbol{\nu}}\newcommand{\bixi}{\boldsymbol{\xi}}\newcommand{\biomicron}{\boldsymbol{\micron}}\newcommand{\bipi}{\boldsymbol{\pi}}\newcommand{\birho}{\boldsymbol{\rho}}\newcommand{\bisigma}{\boldsymbol{\sigma}}\newcommand{\bitau}{\boldsymbol{\tau}}\newcommand{\biupsilon}{\boldsymbol{\upsilon}}\newcommand{\biphi}{\boldsymbol{\phi}}\newcommand{\bichi}{\boldsymbol{\chi}}\newcommand{\bipsi}{\boldsymbol{\psi}}\newcommand{\biomega}{\boldsymbol{\omega}}\( \vec N \) is the density of retinal nerve fibers (unit: number/mm^2^)
\newcommand{\bialpha}{\boldsymbol{\alpha}}\newcommand{\bibeta}{\boldsymbol{\beta}}\newcommand{\bigamma}{\boldsymbol{\gamma}}\newcommand{\bidelta}{\boldsymbol{\delta}}\newcommand{\bivarepsilon}{\boldsymbol{\varepsilon}}\newcommand{\bizeta}{\boldsymbol{\zeta}}\newcommand{\bieta}{\boldsymbol{\eta}}\newcommand{\bitheta}{\boldsymbol{\theta}}\newcommand{\biiota}{\boldsymbol{\iota}}\newcommand{\bikappa}{\boldsymbol{\kappa}}\newcommand{\bilambda}{\boldsymbol{\lambda}}\newcommand{\bimu}{\boldsymbol{\mu}}\newcommand{\binu}{\boldsymbol{\nu}}\newcommand{\bixi}{\boldsymbol{\xi}}\newcommand{\biomicron}{\boldsymbol{\micron}}\newcommand{\bipi}{\boldsymbol{\pi}}\newcommand{\birho}{\boldsymbol{\rho}}\newcommand{\bisigma}{\boldsymbol{\sigma}}\newcommand{\bitau}{\boldsymbol{\tau}}\newcommand{\biupsilon}{\boldsymbol{\upsilon}}\newcommand{\biphi}{\boldsymbol{\phi}}\newcommand{\bichi}{\boldsymbol{\chi}}\newcommand{\bipsi}{\boldsymbol{\psi}}\newcommand{\biomega}{\boldsymbol{\omega}}\(d \vec l \) is a differential line element of the sample circle

The magnitude of \newcommand{\bialpha}{\boldsymbol{\alpha}}\newcommand{\bibeta}{\boldsymbol{\beta}}\newcommand{\bigamma}{\boldsymbol{\gamma}}\newcommand{\bidelta}{\boldsymbol{\delta}}\newcommand{\bivarepsilon}{\boldsymbol{\varepsilon}}\newcommand{\bizeta}{\boldsymbol{\zeta}}\newcommand{\bieta}{\boldsymbol{\eta}}\newcommand{\bitheta}{\boldsymbol{\theta}}\newcommand{\biiota}{\boldsymbol{\iota}}\newcommand{\bikappa}{\boldsymbol{\kappa}}\newcommand{\bilambda}{\boldsymbol{\lambda}}\newcommand{\bimu}{\boldsymbol{\mu}}\newcommand{\binu}{\boldsymbol{\nu}}\newcommand{\bixi}{\boldsymbol{\xi}}\newcommand{\biomicron}{\boldsymbol{\micron}}\newcommand{\bipi}{\boldsymbol{\pi}}\newcommand{\birho}{\boldsymbol{\rho}}\newcommand{\bisigma}{\boldsymbol{\sigma}}\newcommand{\bitau}{\boldsymbol{\tau}}\newcommand{\biupsilon}{\boldsymbol{\upsilon}}\newcommand{\biphi}{\boldsymbol{\phi}}\newcommand{\bichi}{\boldsymbol{\chi}}\newcommand{\bipsi}{\boldsymbol{\psi}}\newcommand{\biomega}{\boldsymbol{\omega}}\(a \vec N \) is equal to the NFL thickness if we define \newcommand{\bialpha}{\boldsymbol{\alpha}}\newcommand{\bibeta}{\boldsymbol{\beta}}\newcommand{\bigamma}{\boldsymbol{\gamma}}\newcommand{\bidelta}{\boldsymbol{\delta}}\newcommand{\bivarepsilon}{\boldsymbol{\varepsilon}}\newcommand{\bizeta}{\boldsymbol{\zeta}}\newcommand{\bieta}{\boldsymbol{\eta}}\newcommand{\bitheta}{\boldsymbol{\theta}}\newcommand{\biiota}{\boldsymbol{\iota}}\newcommand{\bikappa}{\boldsymbol{\kappa}}\newcommand{\bilambda}{\boldsymbol{\lambda}}\newcommand{\bimu}{\boldsymbol{\mu}}\newcommand{\binu}{\boldsymbol{\nu}}\newcommand{\bixi}{\boldsymbol{\xi}}\newcommand{\biomicron}{\boldsymbol{\micron}}\newcommand{\bipi}{\boldsymbol{\pi}}\newcommand{\birho}{\boldsymbol{\rho}}\newcommand{\bisigma}{\boldsymbol{\sigma}}\newcommand{\bitau}{\boldsymbol{\tau}}\newcommand{\biupsilon}{\boldsymbol{\upsilon}}\newcommand{\biphi}{\boldsymbol{\phi}}\newcommand{\bichi}{\boldsymbol{\chi}}\newcommand{\bipsi}{\boldsymbol{\psi}}\newcommand{\biomega}{\boldsymbol{\omega}}\(\Phi \)=\newcommand{\bialpha}{\boldsymbol{\alpha}}\newcommand{\bibeta}{\boldsymbol{\beta}}\newcommand{\bigamma}{\boldsymbol{\gamma}}\newcommand{\bidelta}{\boldsymbol{\delta}}\newcommand{\bivarepsilon}{\boldsymbol{\varepsilon}}\newcommand{\bizeta}{\boldsymbol{\zeta}}\newcommand{\bieta}{\boldsymbol{\eta}}\newcommand{\bitheta}{\boldsymbol{\theta}}\newcommand{\biiota}{\boldsymbol{\iota}}\newcommand{\bikappa}{\boldsymbol{\kappa}}\newcommand{\bilambda}{\boldsymbol{\lambda}}\newcommand{\bimu}{\boldsymbol{\mu}}\newcommand{\binu}{\boldsymbol{\nu}}\newcommand{\bixi}{\boldsymbol{\xi}}\newcommand{\biomicron}{\boldsymbol{\micron}}\newcommand{\bipi}{\boldsymbol{\pi}}\newcommand{\birho}{\boldsymbol{\rho}}\newcommand{\bisigma}{\boldsymbol{\sigma}}\newcommand{\bitau}{\boldsymbol{\tau}}\newcommand{\biupsilon}{\boldsymbol{\upsilon}}\newcommand{\biphi}{\boldsymbol{\phi}}\newcommand{\bichi}{\boldsymbol{\chi}}\newcommand{\bipsi}{\boldsymbol{\psi}}\newcommand{\biomega}{\boldsymbol{\omega}}\({A_{NFL}}\) and calculate the differential of both sides of the above equation

\newcommand{\bialpha}{\boldsymbol{\alpha}}\newcommand{\bibeta}{\boldsymbol{\beta}}\newcommand{\bigamma}{\boldsymbol{\gamma}}\newcommand{\bidelta}{\boldsymbol{\delta}}\newcommand{\bivarepsilon}{\boldsymbol{\varepsilon}}\newcommand{\bizeta}{\boldsymbol{\zeta}}\newcommand{\bieta}{\boldsymbol{\eta}}\newcommand{\bitheta}{\boldsymbol{\theta}}\newcommand{\biiota}{\boldsymbol{\iota}}\newcommand{\bikappa}{\boldsymbol{\kappa}}\newcommand{\bilambda}{\boldsymbol{\lambda}}\newcommand{\bimu}{\boldsymbol{\mu}}\newcommand{\binu}{\boldsymbol{\nu}}\newcommand{\bixi}{\boldsymbol{\xi}}\newcommand{\biomicron}{\boldsymbol{\micron}}\newcommand{\bipi}{\boldsymbol{\pi}}\newcommand{\birho}{\boldsymbol{\rho}}\newcommand{\bisigma}{\boldsymbol{\sigma}}\newcommand{\bitau}{\boldsymbol{\tau}}\newcommand{\biupsilon}{\boldsymbol{\upsilon}}\newcommand{\biphi}{\boldsymbol{\phi}}\newcommand{\bichi}{\boldsymbol{\chi}}\newcommand{\bipsi}{\boldsymbol{\psi}}\newcommand{\biomega}{\boldsymbol{\omega}}\begin{equation}\tag{A4}d\Phi = \vec T \cdot d \vec l = T\cos\beta \,dl \end{equation}

where

\newcommand{\bialpha}{\boldsymbol{\alpha}}\newcommand{\bibeta}{\boldsymbol{\beta}}\newcommand{\bigamma}{\boldsymbol{\gamma}}\newcommand{\bidelta}{\boldsymbol{\delta}}\newcommand{\bivarepsilon}{\boldsymbol{\varepsilon}}\newcommand{\bizeta}{\boldsymbol{\zeta}}\newcommand{\bieta}{\boldsymbol{\eta}}\newcommand{\bitheta}{\boldsymbol{\theta}}\newcommand{\biiota}{\boldsymbol{\iota}}\newcommand{\bikappa}{\boldsymbol{\kappa}}\newcommand{\bilambda}{\boldsymbol{\lambda}}\newcommand{\bimu}{\boldsymbol{\mu}}\newcommand{\binu}{\boldsymbol{\nu}}\newcommand{\bixi}{\boldsymbol{\xi}}\newcommand{\biomicron}{\boldsymbol{\micron}}\newcommand{\bipi}{\boldsymbol{\pi}}\newcommand{\birho}{\boldsymbol{\rho}}\newcommand{\bisigma}{\boldsymbol{\sigma}}\newcommand{\bitau}{\boldsymbol{\tau}}\newcommand{\biupsilon}{\boldsymbol{\upsilon}}\newcommand{\biphi}{\boldsymbol{\phi}}\newcommand{\bichi}{\boldsymbol{\chi}}\newcommand{\bipsi}{\boldsymbol{\psi}}\newcommand{\biomega}{\boldsymbol{\omega}}\(d\Phi \) is the NFF, which is proportional to the number of nerve fibers through a small segment of the sampling circle
\newcommand{\bialpha}{\boldsymbol{\alpha}}\newcommand{\bibeta}{\boldsymbol{\beta}}\newcommand{\bigamma}{\boldsymbol{\gamma}}\newcommand{\bidelta}{\boldsymbol{\delta}}\newcommand{\bivarepsilon}{\boldsymbol{\varepsilon}}\newcommand{\bizeta}{\boldsymbol{\zeta}}\newcommand{\bieta}{\boldsymbol{\eta}}\newcommand{\bitheta}{\boldsymbol{\theta}}\newcommand{\biiota}{\boldsymbol{\iota}}\newcommand{\bikappa}{\boldsymbol{\kappa}}\newcommand{\bilambda}{\boldsymbol{\lambda}}\newcommand{\bimu}{\boldsymbol{\mu}}\newcommand{\binu}{\boldsymbol{\nu}}\newcommand{\bixi}{\boldsymbol{\xi}}\newcommand{\biomicron}{\boldsymbol{\micron}}\newcommand{\bipi}{\boldsymbol{\pi}}\newcommand{\birho}{\boldsymbol{\rho}}\newcommand{\bisigma}{\boldsymbol{\sigma}}\newcommand{\bitau}{\boldsymbol{\tau}}\newcommand{\biupsilon}{\boldsymbol{\upsilon}}\newcommand{\biphi}{\boldsymbol{\phi}}\newcommand{\bichi}{\boldsymbol{\chi}}\newcommand{\bipsi}{\boldsymbol{\psi}}\newcommand{\biomega}{\boldsymbol{\omega}}\( \vec T \) is a vector with the magnitude of the NFL thickness and direction of the nerve fiber trajectory
\newcommand{\bialpha}{\boldsymbol{\alpha}}\newcommand{\bibeta}{\boldsymbol{\beta}}\newcommand{\bigamma}{\boldsymbol{\gamma}}\newcommand{\bidelta}{\boldsymbol{\delta}}\newcommand{\bivarepsilon}{\boldsymbol{\varepsilon}}\newcommand{\bizeta}{\boldsymbol{\zeta}}\newcommand{\bieta}{\boldsymbol{\eta}}\newcommand{\bitheta}{\boldsymbol{\theta}}\newcommand{\biiota}{\boldsymbol{\iota}}\newcommand{\bikappa}{\boldsymbol{\kappa}}\newcommand{\bilambda}{\boldsymbol{\lambda}}\newcommand{\bimu}{\boldsymbol{\mu}}\newcommand{\binu}{\boldsymbol{\nu}}\newcommand{\bixi}{\boldsymbol{\xi}}\newcommand{\biomicron}{\boldsymbol{\micron}}\newcommand{\bipi}{\boldsymbol{\pi}}\newcommand{\birho}{\boldsymbol{\rho}}\newcommand{\bisigma}{\boldsymbol{\sigma}}\newcommand{\bitau}{\boldsymbol{\tau}}\newcommand{\biupsilon}{\boldsymbol{\upsilon}}\newcommand{\biphi}{\boldsymbol{\phi}}\newcommand{\bichi}{\boldsymbol{\chi}}\newcommand{\bipsi}{\boldsymbol{\psi}}\newcommand{\biomega}{\boldsymbol{\omega}}\(d \vec l \) is a differential line segment of the sampling circle
\newcommand{\bialpha}{\boldsymbol{\alpha}}\newcommand{\bibeta}{\boldsymbol{\beta}}\newcommand{\bigamma}{\boldsymbol{\gamma}}\newcommand{\bidelta}{\boldsymbol{\delta}}\newcommand{\bivarepsilon}{\boldsymbol{\varepsilon}}\newcommand{\bizeta}{\boldsymbol{\zeta}}\newcommand{\bieta}{\boldsymbol{\eta}}\newcommand{\bitheta}{\boldsymbol{\theta}}\newcommand{\biiota}{\boldsymbol{\iota}}\newcommand{\bikappa}{\boldsymbol{\kappa}}\newcommand{\bilambda}{\boldsymbol{\lambda}}\newcommand{\bimu}{\boldsymbol{\mu}}\newcommand{\binu}{\boldsymbol{\nu}}\newcommand{\bixi}{\boldsymbol{\xi}}\newcommand{\biomicron}{\boldsymbol{\micron}}\newcommand{\bipi}{\boldsymbol{\pi}}\newcommand{\birho}{\boldsymbol{\rho}}\newcommand{\bisigma}{\boldsymbol{\sigma}}\newcommand{\bitau}{\boldsymbol{\tau}}\newcommand{\biupsilon}{\boldsymbol{\upsilon}}\newcommand{\biphi}{\boldsymbol{\phi}}\newcommand{\bichi}{\boldsymbol{\chi}}\newcommand{\bipsi}{\boldsymbol{\psi}}\newcommand{\biomega}{\boldsymbol{\omega}}\(\beta \) is the skew angle deviating from the perpendicular

Thus, NFL thickness is analogous to electric field strength, and nerve fibers are analogous to electric flux liness. One can think of the integral in Equation A3 as representing the total perpendicular cross-sectional area of all nerve fibers exiting the optic nerve. In an electric field, the flux lines terminate at charges, such as electrons. For the NFF, one can think of the fibers as terminating on RGC. The conservation of NFF in the sampling circles arises from fact that there is a fixed number of nerve fibers in the optic nerve. The total NFF crossing two sampling circles of different diameters should be equal to the number of RGCs in the annular region between them. Given that RGCs are sparse around the disc and represent a negligible proportion of all RGC's, then the flux should be approximately equal between the sampling circles. However, this approximation cannot be extended far into the macula because of the high concentration of RGC's in the macula.

Because the nerve fiber is a three-dimesional structure, \newcommand{\bialpha}{\boldsymbol{\alpha}}\newcommand{\bibeta}{\boldsymbol{\beta}}\newcommand{\bigamma}{\boldsymbol{\gamma}}\newcommand{\bidelta}{\boldsymbol{\delta}}\newcommand{\bivarepsilon}{\boldsymbol{\varepsilon}}\newcommand{\bizeta}{\boldsymbol{\zeta}}\newcommand{\bieta}{\boldsymbol{\eta}}\newcommand{\bitheta}{\boldsymbol{\theta}}\newcommand{\biiota}{\boldsymbol{\iota}}\newcommand{\bikappa}{\boldsymbol{\kappa}}\newcommand{\bilambda}{\boldsymbol{\lambda}}\newcommand{\bimu}{\boldsymbol{\mu}}\newcommand{\binu}{\boldsymbol{\nu}}\newcommand{\bixi}{\boldsymbol{\xi}}\newcommand{\biomicron}{\boldsymbol{\micron}}\newcommand{\bipi}{\boldsymbol{\pi}}\newcommand{\birho}{\boldsymbol{\rho}}\newcommand{\bisigma}{\boldsymbol{\sigma}}\newcommand{\bitau}{\boldsymbol{\tau}}\newcommand{\biupsilon}{\boldsymbol{\upsilon}}\newcommand{\biphi}{\boldsymbol{\phi}}\newcommand{\bichi}{\boldsymbol{\chi}}\newcommand{\bipsi}{\boldsymbol{\psi}}\newcommand{\biomega}{\boldsymbol{\omega}}\(\beta \) could have transverse components in the x-y plane and an axial component z-plane. However, the axial component is zero because we measured the NFL thickness perpendicular to the inner retinal surface. Therefore the problem is simplified to two dimensions.

To extend this method to macular area, we compensate for the flux in the macular area. The compensation is related to the nerve fibers that end in RGCs. For each track, we can assume the total number of nerve fibers at the disc edge is proportional to all RGCs on the track. In the macular area, nerve fibers started in the midline and ended at disc edge. The nerve fiber number is equal to the integration of ganglion cell numbers along the track

\newcommand{\bialpha}{\boldsymbol{\alpha}}\newcommand{\bibeta}{\boldsymbol{\beta}}\newcommand{\bigamma}{\boldsymbol{\gamma}}\newcommand{\bidelta}{\boldsymbol{\delta}}\newcommand{\bivarepsilon}{\boldsymbol{\varepsilon}}\newcommand{\bizeta}{\boldsymbol{\zeta}}\newcommand{\bieta}{\boldsymbol{\eta}}\newcommand{\bitheta}{\boldsymbol{\theta}}\newcommand{\biiota}{\boldsymbol{\iota}}\newcommand{\bikappa}{\boldsymbol{\kappa}}\newcommand{\bilambda}{\boldsymbol{\lambda}}\newcommand{\bimu}{\boldsymbol{\mu}}\newcommand{\binu}{\boldsymbol{\nu}}\newcommand{\bixi}{\boldsymbol{\xi}}\newcommand{\biomicron}{\boldsymbol{\micron}}\newcommand{\bipi}{\boldsymbol{\pi}}\newcommand{\birho}{\boldsymbol{\rho}}\newcommand{\bisigma}{\boldsymbol{\sigma}}\newcommand{\bitau}{\boldsymbol{\tau}}\newcommand{\biupsilon}{\boldsymbol{\upsilon}}\newcommand{\biphi}{\boldsymbol{\phi}}\newcommand{\bichi}{\boldsymbol{\chi}}\newcommand{\bipsi}{\boldsymbol{\psi}}\newcommand{\biomega}{\boldsymbol{\omega}}\begin{equation}\tag{A5}N(s) = \mathop \int \nolimits_0^s \varepsilon RGCw\,ds{\rm }\end{equation}

where *N* is the nerve fiber number, \newcommand{\bialpha}{\boldsymbol{\alpha}}\newcommand{\bibeta}{\boldsymbol{\beta}}\newcommand{\bigamma}{\boldsymbol{\gamma}}\newcommand{\bidelta}{\boldsymbol{\delta}}\newcommand{\bivarepsilon}{\boldsymbol{\varepsilon}}\newcommand{\bizeta}{\boldsymbol{\zeta}}\newcommand{\bieta}{\boldsymbol{\eta}}\newcommand{\bitheta}{\boldsymbol{\theta}}\newcommand{\biiota}{\boldsymbol{\iota}}\newcommand{\bikappa}{\boldsymbol{\kappa}}\newcommand{\bilambda}{\boldsymbol{\lambda}}\newcommand{\bimu}{\boldsymbol{\mu}}\newcommand{\binu}{\boldsymbol{\nu}}\newcommand{\bixi}{\boldsymbol{\xi}}\newcommand{\biomicron}{\boldsymbol{\micron}}\newcommand{\bipi}{\boldsymbol{\pi}}\newcommand{\birho}{\boldsymbol{\rho}}\newcommand{\bisigma}{\boldsymbol{\sigma}}\newcommand{\bitau}{\boldsymbol{\tau}}\newcommand{\biupsilon}{\boldsymbol{\upsilon}}\newcommand{\biphi}{\boldsymbol{\phi}}\newcommand{\bichi}{\boldsymbol{\chi}}\newcommand{\bipsi}{\boldsymbol{\psi}}\newcommand{\biomega}{\boldsymbol{\omega}}\(\varepsilon \) is the average nerve fiber numbers of one ganglion cell, RGC is the retinal ganglion cell count (1/mm^2^), *w* is the track width, *s* is the distance position from the start location on the track. The integration is along the track, starting from the midline and ending at disc center.

The maximum *N_D_* is obtained at the disc edge **S** = *S_D_*. The ratio between *N_D_* and *N(S)*

\newcommand{\bialpha}{\boldsymbol{\alpha}}\newcommand{\bibeta}{\boldsymbol{\beta}}\newcommand{\bigamma}{\boldsymbol{\gamma}}\newcommand{\bidelta}{\boldsymbol{\delta}}\newcommand{\bivarepsilon}{\boldsymbol{\varepsilon}}\newcommand{\bizeta}{\boldsymbol{\zeta}}\newcommand{\bieta}{\boldsymbol{\eta}}\newcommand{\bitheta}{\boldsymbol{\theta}}\newcommand{\biiota}{\boldsymbol{\iota}}\newcommand{\bikappa}{\boldsymbol{\kappa}}\newcommand{\bilambda}{\boldsymbol{\lambda}}\newcommand{\bimu}{\boldsymbol{\mu}}\newcommand{\binu}{\boldsymbol{\nu}}\newcommand{\bixi}{\boldsymbol{\xi}}\newcommand{\biomicron}{\boldsymbol{\micron}}\newcommand{\bipi}{\boldsymbol{\pi}}\newcommand{\birho}{\boldsymbol{\rho}}\newcommand{\bisigma}{\boldsymbol{\sigma}}\newcommand{\bitau}{\boldsymbol{\tau}}\newcommand{\biupsilon}{\boldsymbol{\upsilon}}\newcommand{\biphi}{\boldsymbol{\phi}}\newcommand{\bichi}{\boldsymbol{\chi}}\newcommand{\bipsi}{\boldsymbol{\psi}}\newcommand{\biomega}{\boldsymbol{\omega}}\begin{equation}\tag{A6}{{{N_D}} \over {N(S)}} = {{\mathop \int_0^{{s_D}} RGCw\,ds} \over {\mathop \int_0^s RGCw\,ds}}{\rm }\end{equation}

*N* is proportional to the flux. Thus, the flux for tracks in the macula is compensated by the known distribution of RGCs. With this compensation, the flux is approximately conserved along the track. For calculation convenience, we used the disc center as the coordinate origin and starting point of all tracks. The compensation \newcommand{\bialpha}{\boldsymbol{\alpha}}\newcommand{\bibeta}{\boldsymbol{\beta}}\newcommand{\bigamma}{\boldsymbol{\gamma}}\newcommand{\bidelta}{\boldsymbol{\delta}}\newcommand{\bivarepsilon}{\boldsymbol{\varepsilon}}\newcommand{\bizeta}{\boldsymbol{\zeta}}\newcommand{\bieta}{\boldsymbol{\eta}}\newcommand{\bitheta}{\boldsymbol{\theta}}\newcommand{\biiota}{\boldsymbol{\iota}}\newcommand{\bikappa}{\boldsymbol{\kappa}}\newcommand{\bilambda}{\boldsymbol{\lambda}}\newcommand{\bimu}{\boldsymbol{\mu}}\newcommand{\binu}{\boldsymbol{\nu}}\newcommand{\bixi}{\boldsymbol{\xi}}\newcommand{\biomicron}{\boldsymbol{\micron}}\newcommand{\bipi}{\boldsymbol{\pi}}\newcommand{\birho}{\boldsymbol{\rho}}\newcommand{\bisigma}{\boldsymbol{\sigma}}\newcommand{\bitau}{\boldsymbol{\tau}}\newcommand{\biupsilon}{\boldsymbol{\upsilon}}\newcommand{\biphi}{\boldsymbol{\phi}}\newcommand{\bichi}{\boldsymbol{\chi}}\newcommand{\bipsi}{\boldsymbol{\psi}}\newcommand{\biomega}{\boldsymbol{\omega}}\({\gamma _n}\) can rewritten as

\newcommand{\bialpha}{\boldsymbol{\alpha}}\newcommand{\bibeta}{\boldsymbol{\beta}}\newcommand{\bigamma}{\boldsymbol{\gamma}}\newcommand{\bidelta}{\boldsymbol{\delta}}\newcommand{\bivarepsilon}{\boldsymbol{\varepsilon}}\newcommand{\bizeta}{\boldsymbol{\zeta}}\newcommand{\bieta}{\boldsymbol{\eta}}\newcommand{\bitheta}{\boldsymbol{\theta}}\newcommand{\biiota}{\boldsymbol{\iota}}\newcommand{\bikappa}{\boldsymbol{\kappa}}\newcommand{\bilambda}{\boldsymbol{\lambda}}\newcommand{\bimu}{\boldsymbol{\mu}}\newcommand{\binu}{\boldsymbol{\nu}}\newcommand{\bixi}{\boldsymbol{\xi}}\newcommand{\biomicron}{\boldsymbol{\micron}}\newcommand{\bipi}{\boldsymbol{\pi}}\newcommand{\birho}{\boldsymbol{\rho}}\newcommand{\bisigma}{\boldsymbol{\sigma}}\newcommand{\bitau}{\boldsymbol{\tau}}\newcommand{\biupsilon}{\boldsymbol{\upsilon}}\newcommand{\biphi}{\boldsymbol{\phi}}\newcommand{\bichi}{\boldsymbol{\chi}}\newcommand{\bipsi}{\boldsymbol{\psi}}\newcommand{\biomega}{\boldsymbol{\omega}}\begin{equation}\tag{A7}{\gamma _{n(R)}} = {{\mathop \smallint \nolimits_0^{{R_{max}}} RGCw\cos\alpha\, dr} \over {\mathop \int_0^{{R_{max}}} RGCw\cos\alpha \,dr - \mathop \int_0^R RGCw\cos\alpha\, dr}}{\rm }\end{equation}

where \newcommand{\bialpha}{\boldsymbol{\alpha}}\newcommand{\bibeta}{\boldsymbol{\beta}}\newcommand{\bigamma}{\boldsymbol{\gamma}}\newcommand{\bidelta}{\boldsymbol{\delta}}\newcommand{\bivarepsilon}{\boldsymbol{\varepsilon}}\newcommand{\bizeta}{\boldsymbol{\zeta}}\newcommand{\bieta}{\boldsymbol{\eta}}\newcommand{\bitheta}{\boldsymbol{\theta}}\newcommand{\biiota}{\boldsymbol{\iota}}\newcommand{\bikappa}{\boldsymbol{\kappa}}\newcommand{\bilambda}{\boldsymbol{\lambda}}\newcommand{\bimu}{\boldsymbol{\mu}}\newcommand{\binu}{\boldsymbol{\nu}}\newcommand{\bixi}{\boldsymbol{\xi}}\newcommand{\biomicron}{\boldsymbol{\micron}}\newcommand{\bipi}{\boldsymbol{\pi}}\newcommand{\birho}{\boldsymbol{\rho}}\newcommand{\bisigma}{\boldsymbol{\sigma}}\newcommand{\bitau}{\boldsymbol{\tau}}\newcommand{\biupsilon}{\boldsymbol{\upsilon}}\newcommand{\biphi}{\boldsymbol{\phi}}\newcommand{\bichi}{\boldsymbol{\chi}}\newcommand{\bipsi}{\boldsymbol{\psi}}\newcommand{\biomega}{\boldsymbol{\omega}}\({\gamma _{n(R)}}\) is the compensation coefficient at radius *R* for track *n*, *R_max_* is the maximum radius where the track ends, *α* is the angle between the track and the horizontal line at radius *r*, the above equation can also be rewritten as

\newcommand{\bialpha}{\boldsymbol{\alpha}}\newcommand{\bibeta}{\boldsymbol{\beta}}\newcommand{\bigamma}{\boldsymbol{\gamma}}\newcommand{\bidelta}{\boldsymbol{\delta}}\newcommand{\bivarepsilon}{\boldsymbol{\varepsilon}}\newcommand{\bizeta}{\boldsymbol{\zeta}}\newcommand{\bieta}{\boldsymbol{\eta}}\newcommand{\bitheta}{\boldsymbol{\theta}}\newcommand{\biiota}{\boldsymbol{\iota}}\newcommand{\bikappa}{\boldsymbol{\kappa}}\newcommand{\bilambda}{\boldsymbol{\lambda}}\newcommand{\bimu}{\boldsymbol{\mu}}\newcommand{\binu}{\boldsymbol{\nu}}\newcommand{\bixi}{\boldsymbol{\xi}}\newcommand{\biomicron}{\boldsymbol{\micron}}\newcommand{\bipi}{\boldsymbol{\pi}}\newcommand{\birho}{\boldsymbol{\rho}}\newcommand{\bisigma}{\boldsymbol{\sigma}}\newcommand{\bitau}{\boldsymbol{\tau}}\newcommand{\biupsilon}{\boldsymbol{\upsilon}}\newcommand{\biphi}{\boldsymbol{\phi}}\newcommand{\bichi}{\boldsymbol{\chi}}\newcommand{\bipsi}{\boldsymbol{\psi}}\newcommand{\biomega}{\boldsymbol{\omega}}\begin{equation}\tag{A8}{\gamma _{n(R)}} = {1 \over {1 - {{\mathop \int_0^R RGCw\cos\alpha\, dr} \over {\mathop \int_0^{{R_{max}}} RGCw\cos\alpha \,dr}}}}{\rm }\end{equation}

Based on a typical ganglion cell density map^25^ and an averaged nerve fiber trajectory^24^ (Fig. 4A), we calculated the compensation coefficient for each track passing through the macular area (tracks *φ_n_*(*r=0*)=110°∼270°, assuming 0° as the nasal midline) using Equation A8. For the other tracks, we set the compensation to 1 because the compensation is already close to 1 at tracks 110° and 270°. Thus we created a matrix of compensation\newcommand{\bialpha}{\boldsymbol{\alpha}}\newcommand{\bibeta}{\boldsymbol{\beta}}\newcommand{\bigamma}{\boldsymbol{\gamma}}\newcommand{\bidelta}{\boldsymbol{\delta}}\newcommand{\bivarepsilon}{\boldsymbol{\varepsilon}}\newcommand{\bizeta}{\boldsymbol{\zeta}}\newcommand{\bieta}{\boldsymbol{\eta}}\newcommand{\bitheta}{\boldsymbol{\theta}}\newcommand{\biiota}{\boldsymbol{\iota}}\newcommand{\bikappa}{\boldsymbol{\kappa}}\newcommand{\bilambda}{\boldsymbol{\lambda}}\newcommand{\bimu}{\boldsymbol{\mu}}\newcommand{\binu}{\boldsymbol{\nu}}\newcommand{\bixi}{\boldsymbol{\xi}}\newcommand{\biomicron}{\boldsymbol{\micron}}\newcommand{\bipi}{\boldsymbol{\pi}}\newcommand{\birho}{\boldsymbol{\rho}}\newcommand{\bisigma}{\boldsymbol{\sigma}}\newcommand{\bitau}{\boldsymbol{\tau}}\newcommand{\biupsilon}{\boldsymbol{\upsilon}}\newcommand{\biphi}{\boldsymbol{\phi}}\newcommand{\bichi}{\boldsymbol{\chi}}\newcommand{\bipsi}{\boldsymbol{\psi}}\newcommand{\biomega}{\boldsymbol{\omega}}\(\gamma \) (Fig. 4).
